# Cellular Reprogramming and Immortality: Expression Profiling Reveals Putative Genes Involved in *Turritopsis dohrnii’s* Life Cycle Reversal

**DOI:** 10.1093/gbe/evab136

**Published:** 2021-06-16

**Authors:** Yui Matsumoto, Maria Pia Miglietta

**Affiliations:** Department of Marine Biology, Texas A&M University at Galveston, Texas, USA

**Keywords:** reverse development, ontogeny reversal, cell transdifferentiation, RNA-sequencing, differential gene expression

## Abstract

To gather insight on the genetic network of cell reprogramming and reverse development in a nonmodel cnidarian system, we produced and annotated a transcriptome of the hydrozoan *Turritopsis dohrnii*, whose medusae respond to damage or senescence by metamorphosing into a juvenile stage (the polyp), briefly passing through an intermediate and uncharacterized stage (the cyst), where cellular transdifferentiation occurs. We conducted sequential and pairwise differential gene expression (DGE) analyses of the major life cycle stages involved in the ontogenetic reversal of *T. dohrnii.* Our DGE analyses of sequential stages of *T. dohrnii’s* life cycle stages show that novel and characterized genes associated with aging/lifespan, regulation of transposable elements, DNA repair, and damage response, and Ubiquitin-related processes, among others, were enriched in the cyst stage. Our pairwise DGE analyses show that, when compared with the colonial polyp, the medusa is enriched with genes involved in membrane transport, the nervous system, components of the mesoglea, and muscle contraction, whereas genes involved in chitin metabolism and the formation of the primary germ layers are suppressed. The colonial polyp and reversed polyp (from cyst) show significant differences in gene expression. The reversed polyp is enriched with genes involved in processes such as chromatin remodeling and organization, matrix metalloproteinases, and embryonic development whereas suppressing genes involved in RAC G-protein signaling pathways. In summary, we identify genetic networks potentially involved in the reverse development of *T. dohrnii* and produce a transcriptome profile of all its life cycle stages, and paving the way for its use as a system for research on cell reprogramming.

## Introduction

Significance

We investigate the gene expression profiles of life cycle stages of the “Immortal Jellyfish,” *Turritopsis dohrnii*. We find that the cyst, a unique and intermediate stage between a dying medusa and a new polyp, upregulates genes related to aging/lifespan, transposable elements, DNA repair and damage, and several genes of unknown function. We show that polyps, although morphologically identical, show characteristic transcriptome profiling depending on their origin (asexual budding from the colony or reverse development). Overall, we provide insight into genes/transcripts that are responsible for and/or associated with the mechanism of cellular reprogramming that occurs during the reverse development of *T. dohrnii.*

The ultimate goal of regenerative research is to replace damaged cells in response to injuries and aging (Shenoy and Blelloch 2012). Transdifferentiation (or cell reprogramming), a process through which a mature somatic cell transforms into a new type of mature somatic cell (Jopling et al. 2011), can achieve this goal. In vitro reprogramming of somatic cells has proven to be a powerful tool and has allowed the identification of some of the genetic factors required to change identity (Dobrovolskaia et al. 2003; Motoyama et al. 2009; Limbert et al. 2011). However, the mechanisms and molecular drivers by which cells spontaneously (in vivo) leave a differentiated state to become a new lineage are poorly understood (Merrell and Stanger 2016), in large part, because of the difficulties of inducing transdifferentiation in live model systems (Abad et al. 2013; Alvarado and Yamanaka 2014). Additionally, because transdifferentiation in available model systems takes place over weeks, modeling its underlying gene regulatory network is problematic (Kaity et al. 2018). Therefore, it is necessary to identify nontraditional species with relevant life history and physiological traits suited for the investigation of genetic mechanism(s) of cellular stability and plasticity in vivo. 

The discovery of reverse development in the cnidarian *Turritopsis dohrnii* (class Hydrozoa) represents a promising new research system (Bavestrello et al. 1992; Piraino et al. 1996; Miglietta and Lessios 2009). Faced with unfavorable circumstances, the medusa naturally undergoes cellular reprogramming to revert to a younger life cycle stage (the polyp), thus avoiding death indefinitely. Although the majority of hydromedusae become reproductively mature, release gametes, and die, medusae of *T. dohrnii* that are stressed, damaged, or senescent settle on a surface and transform into a cyst stage that, in 24–72 h, metamorphoses back into a single juvenile polyp (Piraino et al. 1996; Schmich et al. 2007; Matsumoto et al. 2019; Miglietta et al. 2019). By asexual reproduction, the polyp can develop into a larger colony (i.e., colonial polyp) that can then release new medusae. Because of its unique life cycle, *T. dohrnii* has been popularized as the “Immortal Jellyfish.”

*Turritopsis*  *dohrnii* is also heavily understudied, with fewer than a dozen papers published since the discovery of its life cycle (Alvarado and Yamanaka 2014). This is mostly due to the difficulties in collecting *T. dohrnii* in the field and in handling the species in the laboratory. However, with its unique potential for rejuvenation and the ability to induce cellular reprogramming under controlled laboratory conditions in approximately 24 h (Piraino et al. 1996; Matsumoto et al. 2019), *T. dohrnii* represents a promising albeit unexplored system to investigate molecular mechanisms of cell stability and regeneration that may be relevant to the development of all other animals, including humans.

Recently, a comparison of three individually assembled de novo transcriptomes of the colonial polyp, cyst, and medusa of *T. dohrnii* has provided preliminary insight on the differences in the number of transcripts (i.e., raw count) annotated with specific Gene Ontology (GO) terms (Matsumoto et al. 2019). Transcripts associated with telomere organization and maintenance, DNA integration, repair, and damage response were among those that showed high expression in the cyst relative to medusa and polyp. Processes associated with cell signaling, division, differentiation, and development showed low expression in the cyst relative to the medusa and polyp. However, the three sequenced libraries (polyp, cyst, and medusa) were not assembled into a single annotated transcriptome because the stages originated from different sampling locations, lacked biological replicates, and were sequenced using different platforms. As a result, differential gene expression (DGE) analyses of the life cycle stages were not conducted, and changes in expression levels between the different stages could not be quantified. Matsumoto et al. (2019) also did not sample reversed polyps (i.e., polyps that originate from the medusa through reverse development). Finally, differences in gene activity between the colonial polyp and medusa stage, and polyps developed from different developmental processes, namely asexual budding and reverse development, have yet to be explored.

We investigate the sequential changes in gene expression that occur during the reverse development of *T. dohrnii* (from colonial polyp to medusa, to cyst, to reversed polyp), with an emphasis on genes that are upregulated in the cyst, where cellular transdifferentiation occurs. We also conduct a pairwise DGE analysis to compare the transcriptional profiles of the benthic colonial polyp and planktonic medusa, and those of the colonial polyp, developed in the wild through asexual budding within a larger colony, and the reversed polyp, developed from the cyst through cellular reprogramming. These analyses reveal differences in gene activity between ecologically and morphologically different stages (benthic and colonial polyp vs. planktonic and solitary medusa) and between morphologically similar stages (polyps) generated by two very different developmental pathways, asexual budding and reverse development.

In summary, this research aims to clarify the genetic pathways involved in the reverse development and cell transdifferentiation of *T. dohrnii* through sequential and pairwise transcriptomic comparison of life cycle stages involved in both forward and reverse development. These genomic tools will further the potential of *T. dohrnii* as a system for the study of the mechanisms and molecular drivers by which cells spontaneously leave a differentiated state to become a new lineage.

## Results

### Transcriptome Assembly and Characterization

**Fig. 1.**
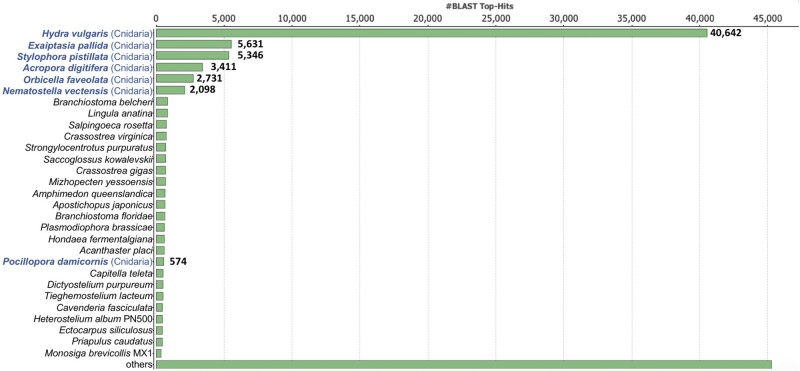
Species distribution of top BLASTx hits in final assembly. Total contigs with BLASTx hit(s): 116,110 transcripts; Blue font: Cnidarian taxa.

The Metazoa benchmarking universal single-copy orthologs (BUSCO) (Simão et al. 2015) analysis reported 97.9% completeness (95.6% complete, 2.2% partial), indicating that our initial transcriptome assembly is highly complete in terms of gene content (supplementary Appendix A, Supplementary Material online). The final transcriptome assembly (∼265.685 Mbp) resulted in 204,031 transcripts and 127,645 unigenes with a GC content of 38.29% (supplementary Appendix B, Supplementary Material online). The N50 of the transcriptome is 1,734 bp with a median contig length of 832 bp and an average length of 1,258.07 bp. Approximately 80% of the transcriptome (162,010 out of 204,031 contigs) was annotated with the following annotation pipelines: B2G (NCBI Nonredundant), InterProScan, COG/EggNOG, KEGG, Rfam, and the Hydrozoa EST; and GO terms were found among ∼42% (85,960 out of 204,031) of contigs of the transcriptome (supplementary Appendix C, Supplementary Material online). The majority of the top-BLAST (basic local alignment search tool) hits belonged to six cnidarian species, *Hydra vulgaris, Exaiptasia pallida*, *Stylophora pistillata*, *Acropora digitifera*, *Orbicella faveolata*, and *Nematostella vectensis*, ranked 1st to 6th, accounting for ∼52% (59,859 out of 116,110) of total hits [fig. 1].

### DGE Analysis of Life Cycle Stages Involved in Reverse Development

**Fig. 2.**
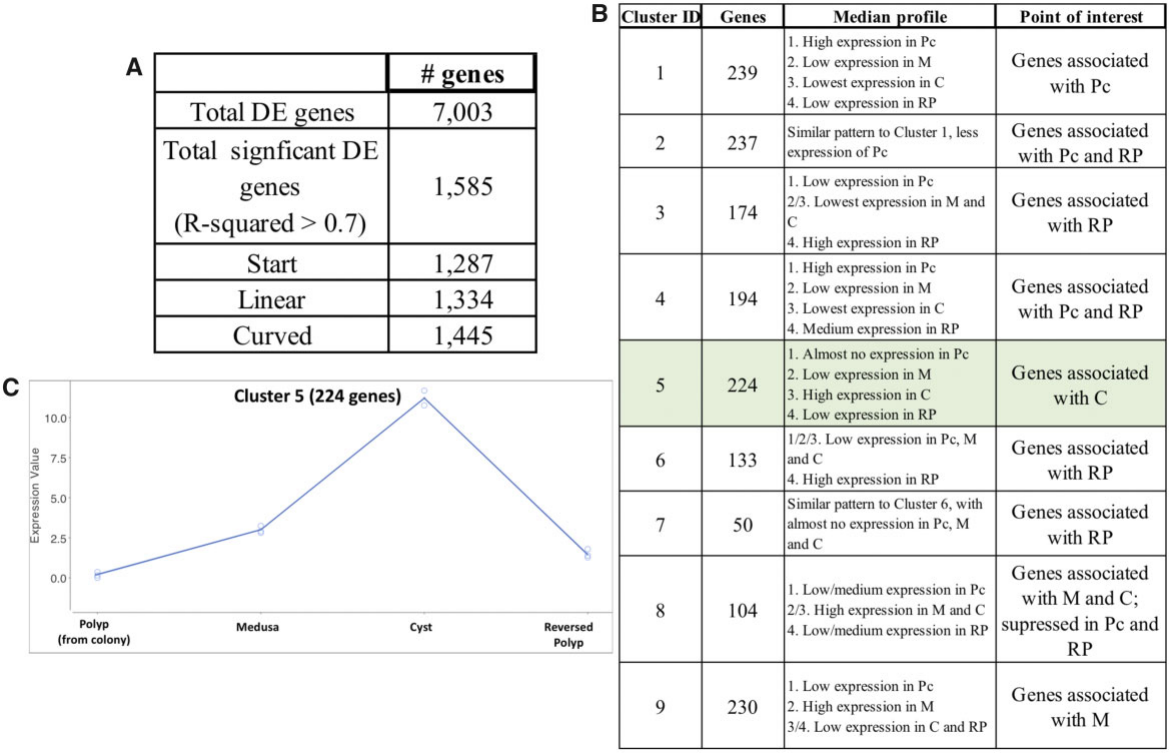
Results from the DGE analysis of *T. dohrnii’*s life cycle reversal. (*A*) Number of significant DEGs. (*B*) Number of significant DE genes in each profile cluster based on hierarchical clustering. (*C*) Averaged gene expression profile of Cluster 5 with 224 DEGs, where expression values that are closer to 0 indicate suppressed genes, larger values indicate enriched genes. P, Polyp (from wild colony); M, Medusa; C, Cyst; RP, Reversed Polyp; Green, Genes associated with Cyst.

**Fig. 3.**
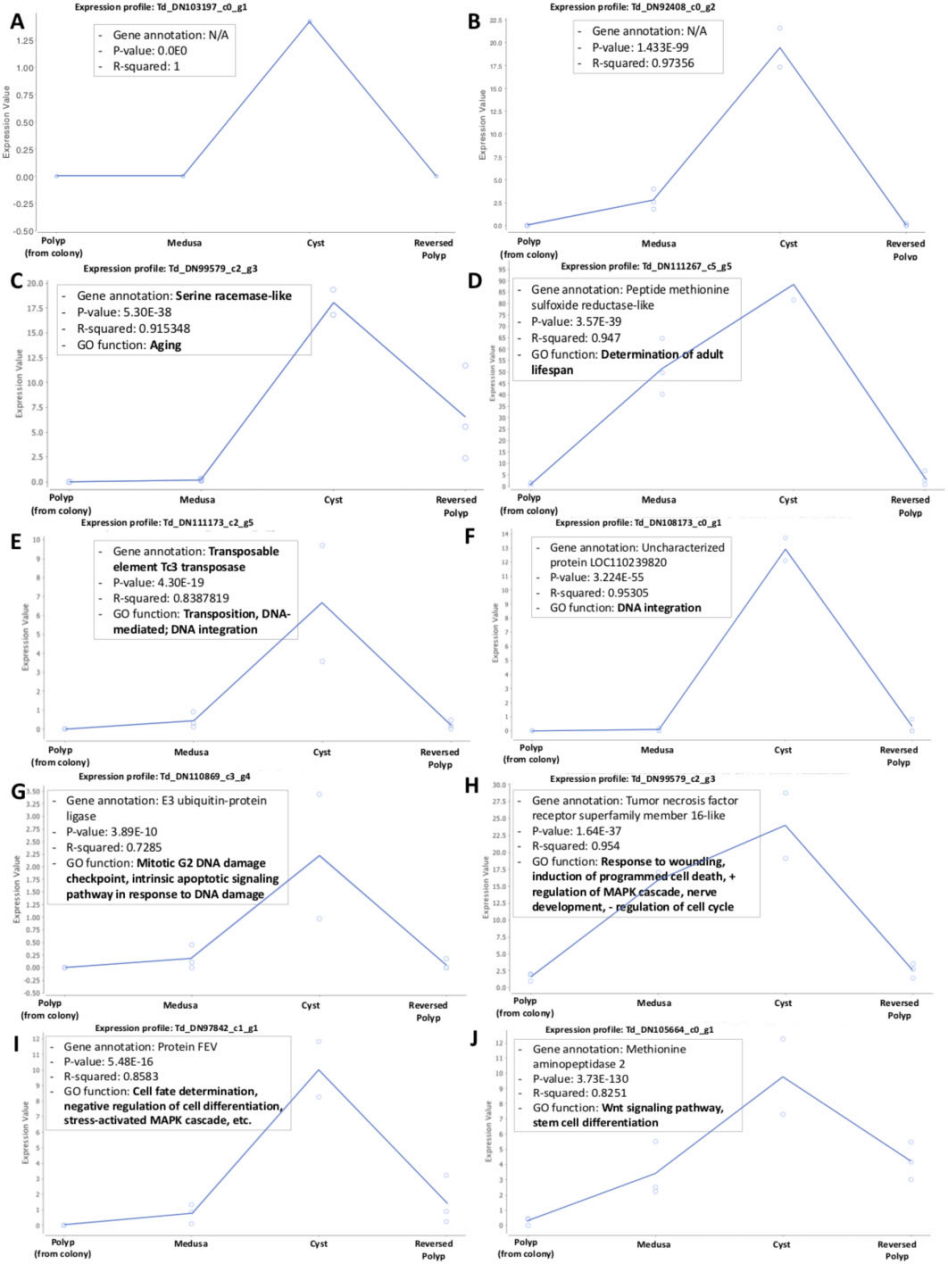
Expression profile of DEGs: (*A*) Expression profile of an unannotated gene Td_DN103197_c0_g1 from cluster 5; (*B*) Expression profile of an unannotated gene Td_DN92408_c0_g2 from cluster 5; (*C*) Serine racemase-like (Td_DN99579_c2_g3); (*D*) Peptide methionine sulfoxide reductase-like (Td_DN111257_c5_g5); (*E*) Transposable element Tc3 Transposase (Td_DN111173_c2_g5); (*F*) Uncharacterized protein LOC110239820 (Td_DN108173_c0_g1); (*G*) E3 Ubiquitin-protein ligase Torpors (Td_DN110869_c3_g4); (*H*) Tumor necrosis factor receptor superfamily member 16-like (Td_DN103087_c0_g3); (*I*) Protein FEV (Td_DN97842_c1_g1); and (*J*) Methionine aminopeptidase 2 (Td_DN105664_c0_g1).

A DGE analysis of the life cycle stages involved in reverse development (Nueda et al. 2014) was performed in the following sequence: 1) Colonial Polyp, 2) Medusa, 3) Cyst, and 4) Reversed Polyp. This sequence reflects the natural ontogenetic trajectory observed in *T. dohrnii* during its life cycle reversal. Overall data set quality was verified with a multidimensional scaling plot (supplementary Appendix D, Supplementary Material online). A total of 7,003 differentially expressed genes (DEGs) were identified, where 1,585 DEGs had *R*^2^ values larger than 0.7 and considered significant for analyses with less than five biological replicates per stage (as recommended by Nueda et al. [2014]) (fig. 2*A*). Nine different gene expression profiles (i.e., clusters) were formed through hierarchical clustering, including 50–239 genes per group (fig. 2*B*; expression profiles in supplementary Appendix E, Supplementary Material online). Among the nine clusters, Cluster 5 with 224 genes represented genes that were exclusive and/or associated to the Cyst stage, reporting a statistically significant increase and subsequent decrease in gene activity during the Medusa to Reversed Polyp transition (fig. 2*B* [Green] and *C*). Cluster 5 showed a higher number of novel genes (i.e., genes with no form of annotation) (26.8%) compared with the overall transcriptome (20.7%), particularly in the top 50 (44%) and 100 (38%) most significantly DEGs (table 1*A*). The 44% among the top 50 DEGs being novel is a striking comparison to the 20.7% of unannotated sequences in the overall transcriptome. The most significant DEG among the 224 genes was a gene with no annotation (Td_DN103197_c0_g1). It had no evidence of any expression in all *T. dohrnii* samples except for the Cyst replicates (fig. 3*A*). Additionally, there were other unannotated genes that showed a significant peak at the Cyst, such as gene Td_DN92408_c0_g2 (fig. 3*B*).

**Table 1:** Annotations of DEGs in Cluster 5 (A) Categories of GO terms and the number of GO terms within category among the top 50, top 100, and all 224 genes. (B) Gene codes, BLAST protein assignment, and GO function (GO term ID) of representative DEGs for each named category. The expression profiles for the listed DEGs are found in figure 3.

	No. of Sequences		
Categories	Top 50	Top 100	All 224 genes
Unannotated (novel genes)	22 (44%)	38 (38%)	60 (26.8%)
Aging and Lifespan	2	4	10
Transposable elements and DNA integration	4	7	10
DNA repair and damage response	0	4	13
Cancer and tumor	3	3	4
Cell and tissue differentiation	2	4	16
Development	5	15	60

Gene Codes	BLAST Protein Assignment	GO function (GO Term ID)	Representative Category
Td_DN103197_c0_g1	N/A	N/A	Unannotated
Td_DN92408_c0_g2	N/A	N/A	Unannotated
Td_DN99579_c2_g3	Serine racemase-like gene	Aging (GO:0007568)	Aging and Lifespan
Td_DN111257_c5_g5	Peptide methionine sulfoxide reductase-like (MsrA)	Determination of adult lifespan (GO:0008340)	Aging and Lifespan
Td_DN111173_c2_g5	Transposable element Tc3 transposase	Transposition, DNA-mediated (GO:0006313)	Transposable elements and DNA integration
		DNA integration (GO:0015074)	
Td_DN108173_c0_g1	Uncharacterized protein LOC110239820	DNA integration (GO:0015074)	Transposable elements and DNA integration
Td_DN110869_c3_g4	E3 Ubiquitin-protein ligase Torpors	Mitotic G2 DNA damage checkpoint (GO:0007095)	DNA repair and damage response
		Intrinsic apoptotic signaling pathway in response to DNA damage (GO:0097193)	
Td_DN103087_c0_g3	Tumor necrosis factor receptor superfamilymember 16-like (TPRG1L)	Response to wounding (GO:0009611)	Cancer and tumor
		Induction of programmed cell death (GO:0012501)	
		Positive regulation of MAPK cascade (GO:0043410)	
		Nerve development (GO:0021675)	
		Negative regulation of cell cycle (GO:0045786)	
Td_DN97842_c1_g1	Protein FEV	Negative regulation of cell differentiation (GO:0045596)	Cell and tissue differentiation
		Cell fate determination (GO:0001709)	
		Stress-activated MAPK cascade (GO:0051403)	
Td_DN105664_c0_g1	Methionine aminopeptidase 2	Wnt signaling pathway (GO:0016055)	Cell and tissue differentiation
		Stem cell differentiation (GO:0048863)	


Note.—MAPK, mitogen-activated protein kinase.

#### Representative DEGs for Each Annotation Category

Besides several unannotated genes (see fig. 3*A* and *B*), genes associated with aging and lifespan, transposable elements, DNA repair and damage response, cancer/tumors, and cell differentiation and development were found in Cluster 5 (table 1 and fig. 3*C–H*). Representative DEGs for each category, with their gene code, BLAST protein assignment, GO function, and term IDs, are reported in detail in table 1*B*. Below we summarize our findings and highlight annotation categories and their representative DEGs found in Cluster 5 that show potential to be involved in the cellular reprogramming and reverse development process in *T. dohrnii*.

We report genes involved in lifespan and aging (i.e., “Serine-racemase-like” and “Peptide methionine sulfoxide reductase-like [*MsrA*])” and in transposition and DNA integration (table 1 and fig. 2*C–F*). Among them is “Transposable element Tc3 transposase,” largely explored in *Caenorhabditis*  *elegans* as one of the most active transposons (Bessereau 2006). Genes associated to DNA repair and response to DNA damage, such as Ubiquitin-related genes, proteins that target cellular destruction by proteasomes in response to DNA damage, were also found in Cluster 5 (table 1 and fig. 2*G*). Several cancer and tumor-related genes were also reported. Among them were “Breast cancer type 1 susceptibility protein isoform X1 *BRCA1*),” “Tumor protein p63-regulated gene 1-like protein (*TPRG1L*),” and “Tumor necrosis factor receptor superfamily member 16-like (*Tnfrsf16*)” (supplementary Appendix F, Supplementary Material online and fig. 2*H*).

Finally, we report numerous genes associated with cell/tissue differentiation and development, such as “Protein PEV” and “Methionine aminopeptidase 2” with functions related to cell fate determination and embryonic development, respectively (table 1 and fig. 3*I* and *J*). A functional gene enrichment analysis of Cluster 5 reported that terms associated with larval development, the response to DNA damage, and protein monoubiquitination were among the most enriched (supplementary Appendix E, Supplementary Material online). On the other hand, processes associated with cytoskeleton and chromosome organization, both specific child-GO terms of broader mitotic cell division processes, were among the most suppressed in Cluster 5 (supplementary Appendix E, Supplementary Material online).

### Pairwise DGE Analyses

**Fig. 4.**
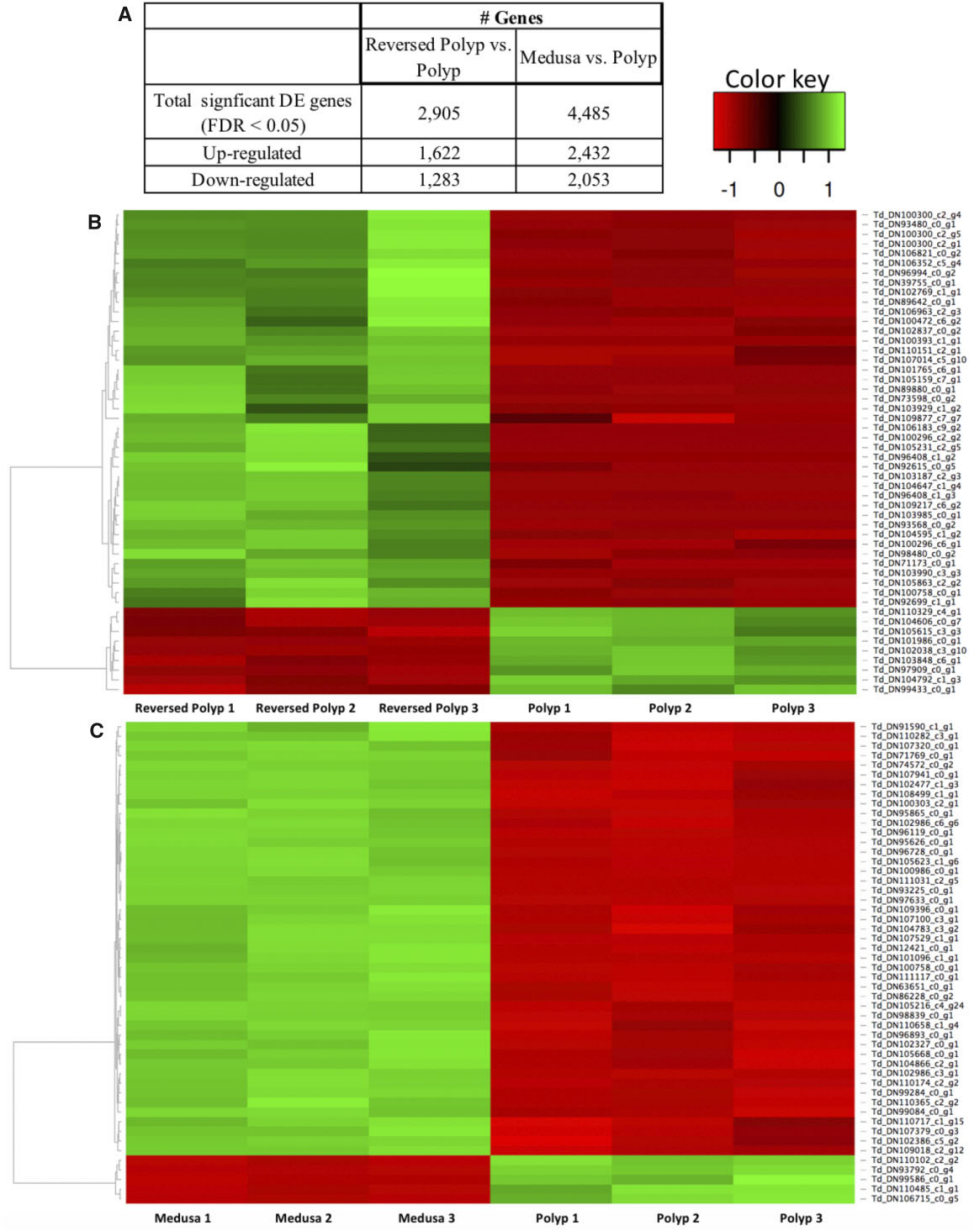
(*A*) Number of overexpressed and underexpressed genes in the Reversed Polyp versus Polyp, and Medusa versus Polyp pairwise DGE analyses. (*B*, *C*) Visualization of the top 50 most DEGs (red: overexpressed, green: underexpressed) when Polyp was compared with the Reversed Polyp and Medusa was compared with the Polyp. Heatmap was created by the Heatmapper software (Babicki et al. 2016).

Pairwise DGE analyses were performed to identify differences in transcriptional profile between 1) Colonial Polyp and Reversed Polyp and 2) Polyp and Medusa. Due to the large number of total DEGs in both comparisons, only the top 50 were further analyzed (fig. 4). DEGs and functional categories that may reflect important physiological differences between the life cycle stages are reported below and summarized in table 2.

**Table 2:** Summary of Pairwise DGE Analyses (Reversed Polyp vs Colonial Polyp; Medusa vs Polyp)

Analysis	Representative Genes	Funtional Category
Reversed Polyp vs Colonial Polyp	Bromodomain adjacent to zinc finger domain protein 1A (BAZ1A) (Oppikofer et al. 2017)	Chromatin remodeling
	Sushi, von Willebrand factor type A, EGF, and pentraxin domain-containing protein 1 (SVEP1) (Shur et al. 2006)	Chromatin remodeling
	Matrix Metalloproteinases (MMPs)-II; MMP-14 isoform X1	MMPs
	Thyrotroph embryonic factor-like (TEF) (Gavriouchkina et al. 2010)	Embryonic development
Medusa vs Polyp	ATP-binding cassette sub-family A member 3-like (ABCA3)	Cellular and transmembrane transport
	Collagen alpha-1, Collagen alpha-6 chain	Collagen
	Clathrin heavy chain (Vassilopoulos et al. 2014)	Muscle development/assembly and contraction
	Actin (Vassilopoulos et al. 2014)	
	Myosin (Rayment et al. 1993)	
	Calmodulin (Chin 2005)	
	Dystroglycan (Adams and Brancaccio 2015)	
	Elongation Factor 1A (Murray et al. 1996)	
	Dystroglycan (Yatsenko and Shcherbata 2014)	Nervous system development
	Clathrin heavy chain (Sato et al. 2009)	

#### Colonial Polyp Versus Reversed Polyp

While sharing 16,614 transcripts, the Reversed Polyp had a larger number of unique transcripts when compared to both the colonial polyp and to the rest of the stages (respectively, 11,603 and 9,729). The majority of the 50 most significant DEGs were enriched in the Reversed Polyp and suppressed in the Colonial Polyp (fig. 4*A* and *B* and supplementary Appendix H, Supplementary Material online). The most enriched gene in the Reversed Polyp was “Bromodomain adjacent to zinc finger domain protein 1A (*BAZ1A*),” followed by “Sushi, von Willebrand factor type A, EGF, and pentraxin domain-containing protein 1 (*SVEP1*)” (supplementary Appendix H, Supplementary Material online), both involved in chromatin remodeling (Wang et al. 2004; Zaghlool et al. 2016). Matrix metalloproteinases (MMPs), such as MMP-II and MMP-14 isoform X1’, were also upregulated in the Reversed Polyp. Furthermore, “Thyrotroph embryonic factor-like(*TEF*)”, a gene active during mammalian embryogenesis (Drolet et al. 1991), with GO terms associated to embryonic development, was overexpressed in the Reversed Polyp (supplementary Appendix H, Supplementary Material online).

**Fig. 5.**
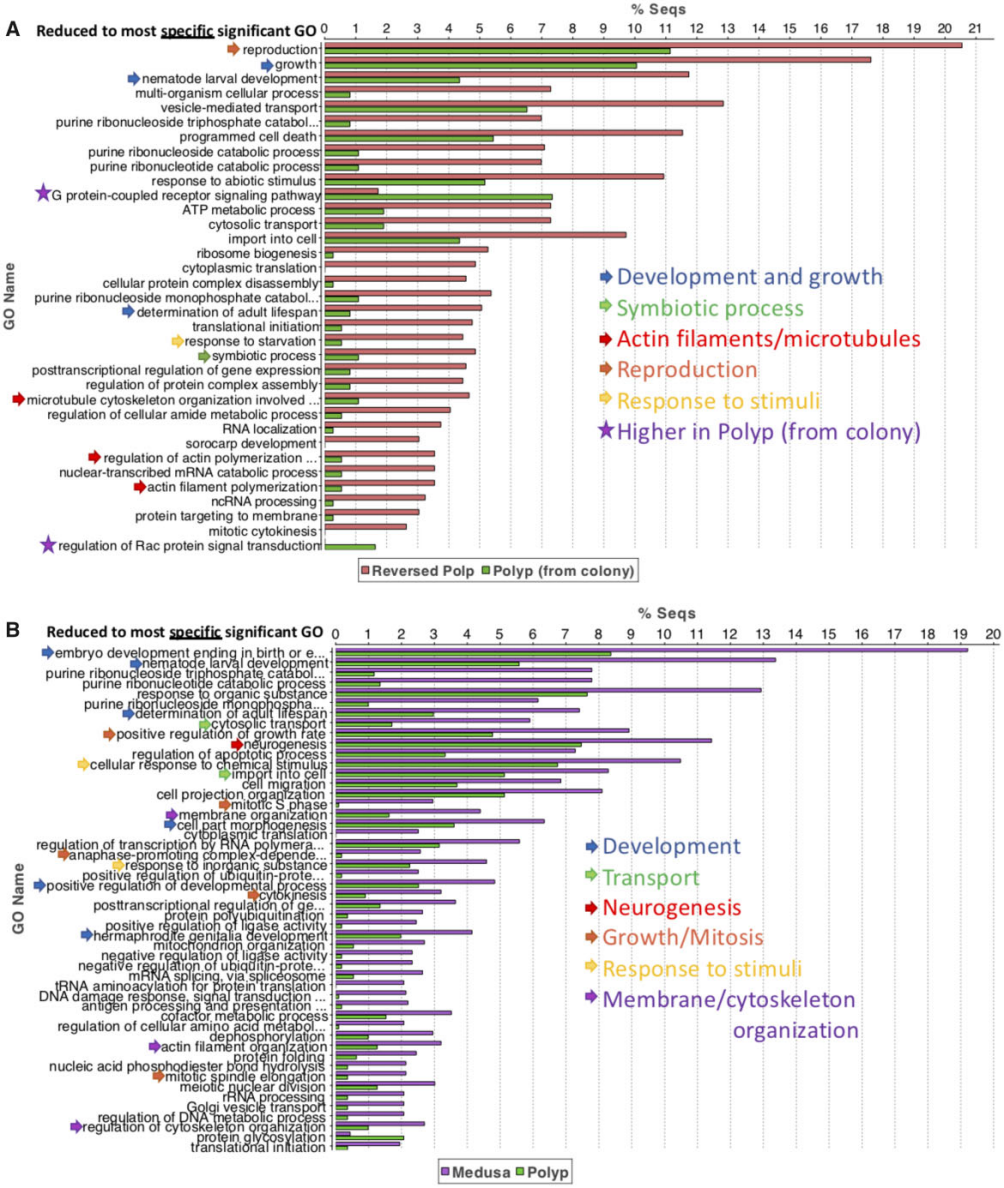
Functional gene enrichment analyses. (*A*) Reversed Polyp versus Polyp and (*B*) Medusa versus Polyp analysis, where significantly enriched and suppressed categories were reduced to the most specific terms (sorted by most different between Reversed Polyp/Medusa and Polyp).

An enrichment analysis using all significant DEGs showed the Reversed Polyp enriched in a variety of GO categories, such as reproduction, development/growth, symbiotic processes, actin filaments/microtubule, and response to stimuli (fig. 5*A*). On the other hand, the Colonial Polyp was enriched in genes associated with the regulation of RAC protein signaling transduction and G-protein receptor signaling pathway (fig. 5*A*), both overlapping processes that use Rho GTP-binding proteins to initiate signaling cascades (Ridley 2006).

##### Medusa Versus Polyp

In the Medusa versus Polyp comparison, the majority of the most significant 50 DEGs were overexpressed in the Medusa, with only three annotated genes overexpressed in the Polyp (i.e., Ectin-like, Galaxin-like, Prefoldin subunit 5 [supplementary Appendix H, Supplementary Material online]). Notably, in the 50 most significant DEGs, 17 (34%) transcripts were novel (supplementary Appendix H, Supplementary Material online). This is substantially higher than the Polyp versus Reversed Polyp comparison that had 10 (20%) novel transcripts (supplementary Appendix H, Supplementary Material online). The most enriched gene in the Medusa was “ATP-binding cassette sub-family A member 3-like (*ABCA3*)” (supplementary Appendix H, Supplementary Material online). In addition to *ABCA3*, five other genes with GO terms involving cellular and transmembrane transport were overexpressed (supplementary Appendix H, Supplementary Material online). Other overexpressed functional categories in the medusa were related to collagen, muscle development/assembly, and contraction, response to external stimuli (light, salt, osmotic stress), glycolysis and glucose catabolism, and nervous system development (table 2 and  supplementary Appendix H, Supplementary Material online). Likewise, an enrichment analysis of all significant DEGs also reported an upregulation of development, transport, nervous system, response to stimuli, and membrane/cytoskeleton organization-related categories in the Medusa (fig. 5*B*). On the other hand, there were only nine GO categories (reduced to most specific) that were found to be enriched in the Polyp (supplementary Appendix I, Supplementary Material online). Among these categories were “Chitin metabolic process,” “Digestion,” and the “Formation of the primary germ layer.”

### Unique and Shared Transcripts among Life Cycle Stages

**Fig. 6.**
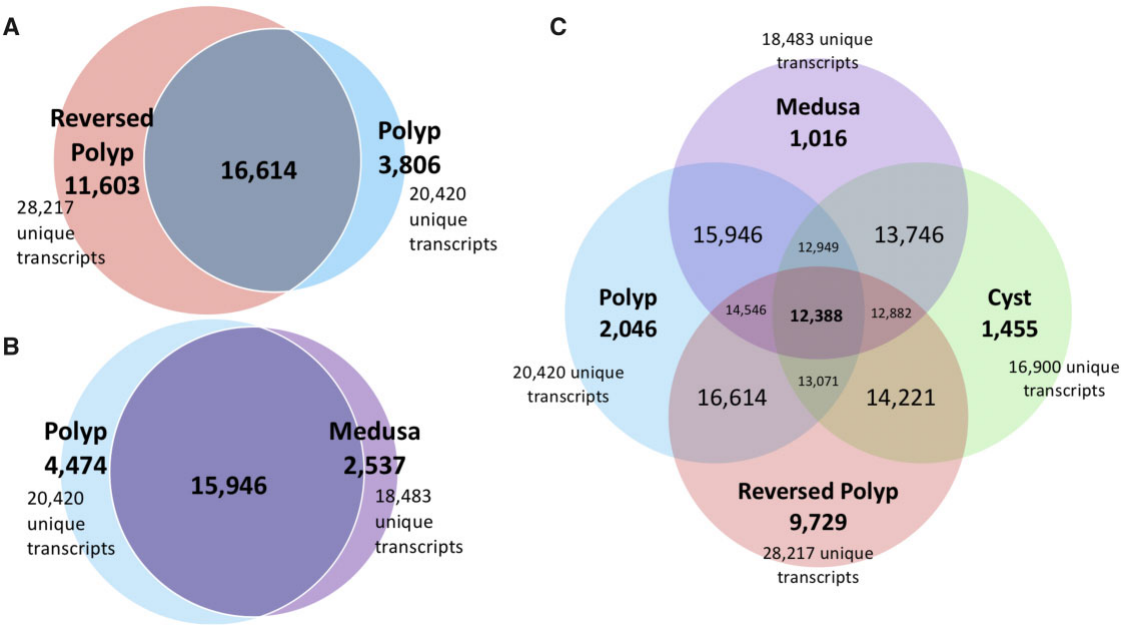
Venn diagrams of BLASTx annotations for shared and unique transcripts. (*A*) Reversed Polyp compared with the Polyp (from colony), (*B*) Medusa compared with the Polyp (from colony), (*C*) All four lifecycle stages involved in reverse development.

Comparison of the BLASTx annotations revealed that the Reversed Polyp and the Colonial Polyp share 16,614 transcripts, and have 11,603 and 3,806 unique transcripts, respectively (fig. 6*A*). The Medusa and Polyp share 15,946 transcripts and have 2,537 and 4,474 unique transcripts, respectively (fig. 6*B*). The four stages involved in life cycle reversal (Polyp, Medusa, Cyst, and Reversed polyp) share 12,388 transcripts, with 2,046 polyp-specific, 1,016 medusa-specific, 1,455 cyst-specific, and 9,729 reversed polyp-specific transcripts (fig. 6*C*).

## Discussion

### Genes Enriched in the Cyst during Reverse Development

The cyst is the focal stage of interest in *T. dohrnii*. The analyses of genes overexpressed in the cyst during life cycle reversal (represented in Cluster 5) help us identify processes directly involved in *T. dohrnii’*s reverse development and cellular reprogramming (i.e., transdifferentiation). We find aging and lifespan-related genes, serine racemase and *MsrA*, are enriched in the cyst. Suppression of serine racemase activity can cause aging-related cognitive dysfunctions in mammals (Turpin et al. 2011), whereas *MsrA* has a role in protecting cells from oxidative damage by destroying reactive intermediates or repairing damaged DNA, subsequently having a profound effect on regulating longevity in mammals (Moskovitz et al. 2001; Weissbach et al. 2002; Koç and Gladyshev 2007). Matsumoto et al. (2019) reported fewer transcripts associated with aging and lifespan GOs in the cyst in comparison to the polyp (colony) and medusa stages. Although the two analyses were different in nature (GO count vs. DEG analyses) and limited comparisons can be made, both indicate that the significant regulation of named genes that influence biological aging and lifespan may be necessary to the reverse development in *T. dohrnii.*

In the cyst, we also found an increased expression of genes controlling transposable element activity, DNA repair, and response to DNA damage, such as Ubiquitin-related factors (fig. 3, table 1). This result indicates that the maintenance and regulation of genome integrity have an important role during the ontogenetic reversal and transdifferentiation processes of *T. dohrnii*. A similar result was found in Matsumoto et al. (2019), where an analysis of the raw GO counts in the DNA repair, integration, and transposition categories showed significantly more transcripts in the cyst than the polyp and the medusa. In addition, GO analyses of the cyst stage (Matsumoto et al. 2019) reported that the cellular response to DNA damage stimulus was the only over-represented term associated with the response to stimuli compared to the polyp and medusa. Our results are consistent, where DNA repair, integration, transposition, and response to the damage being overexpressed categories in the cyst. They represent major focus points for future research on the genetics of regenerative and reprogramming mechanisms in *T. dohrnii*.

We also show that several cancer and tumor-related genes known to control the cell cycle and active during embryonic development (Slamon and Cline 1984; Calvo and Drabkin 2000; Monk and Holding 2001) and tissue regeneration (Beausejour and Campisi 2006; Oviedo and Beane 2009), are enriched in the cyst (fig. 3, table 1). Such genes include tumor suppressor *BRCA1*, which repairs DNA and inhibit cells from dividing erratically (Chen and Parmigiani 2007), and *TPRG1K*, a gene regulated by the TERC component of the telomerase enzyme that can activate the NF-κB (Nuclear Factor kappa B) signaling pathway and controls immune response (Liu et al. 2019). The NF-κB pathway has also been recognized as a vital regulator of the initiation and advancement of cancer (Hoesel and Schmid 2013), and its manipulation has been shown to extend lifespan in mice and halt epigenetic aging (Adler et al. 2007).

In summary, our findings illustrate a scenario where maintenance and regulation of genome integrity, regulation of cell division and proliferation, as well as genes canonically involved in the regulation of aging/lifespan, are active during the ontogenetic reversal and transdifferentiation processes of *T. dohrnii*. Furthermore, we find that 44% of the top 50 genes in Cluster 5 (thus upregulated in the cyst and with little or no expression in the other stages) are novel/nonannotated genes (table 1*A*). This is a significantly higher ratio of novel/not annotated genes than found in the entire transcriptome. Moreover, the most significant DEG among the 224 genes in Cluster 5 has also no annotation. This indicates a further avenue of research where an effort should be spent toward the annotation of such genes that seem to play a crucial role in the events of rejuvenation and cellular transdifferentiation that occur in *T. dohrnii*.

### Colonial Polyp Versus Reversed Polyp

The comparison between the colonial polyp and reversed polyp aims to identify differences in gene activity, if any, between the same life cycle stage (the polyp) produced by two different developmental pathways (budding and reverse development through the cyst). Our pairwise DGE and enrichment analyses show that polyps produced through reverse development and polyps originating from a wild colony via asexual budding show remarkably different transcriptome profiles (see figs. 4*A*, 4*B*, 5*A*, and 6*A*), overall having the most distinct patterns of gene expression among the pairwise comparisons performed in this paper. Below, we highlight the roles of genes associated with chromatin remodeling, MMPs, and embryonic development, which were found among the most significantly enriched in the reversed polyp (table 2).

Chromatin remodeling gene *BAZ1A* repairs damaged chromatin and promotes survival in human cells (Oppikofer et al. 2017), whereas chromatin-binding *SVEP1* is involved in cell adhesion. Polymorphism of the *SVEP1* gene has also been correlated with human longevity (Shur et al. 2006; Yashin et al. 2010). In metazoans, MMPs function to maintain the extracellular matrix and regulate the interaction between cells and matrix during regeneration (Yang and Byant 1994; Bai et al. 2005; Vinarsky et al. 2005; Tseng and Levin 2008). These proteinases are also involved in tissue remodeling and engineering, and play important roles in the regeneration of zebrafish (Bai et al. 2005), newts (Vinarsky et al. 2005), and axolotls (Yang and Byant 1994). Among the genes enriched in the reversed polyp, some are associated with embryonic development, such as *TEF*, which plays a prominent role in activating DNA repair genes in response to stress and damage in zebrafish embryonic cells (Gavriouchkina et al. 2010).

We also show that the colonial polyp is enriched in genes related to the Rho GTP-binding signaling cascades (fig. 5*A*), which is involved in a diversity of processes, including development, metabolic regulation, cellular growth and survival, and changes in the actin cytoskeleton (Neves et al. 2002; Yan and Jin 2012) (fig. 5*A*).

In conclusion, our colonial versus reversed polyp comparison shows that reparative and regenerative processes, which include maintaining chromatin, MMP induced tissue remodeling, and genetic pathways vital to embryonic development, are enriched in the reversed polyp but not in the colonial polyp. Such differences may reflect the fact that reverse development as a whole is a regenerative process that includes the re-establishment of cell types and physical features of the polyp from a different life cycle stage, the medusa.

### Medusa Versus Colonial Polyp

The comparison between medusa and colonial polyp aims to identify differences in genetic networks between the planktonic and solitary stage (medusa) and the colonial stage (polyp). Medusae are considered more complex than polyps, being able to swim, sexually reproduce, and having nerves and sensory organs. Morphologically, medusae have a thick mesoglea, whereas polyps have chitinous protective exteriors. We discuss genes and networks attributable to the physiological differences of the medusa and polyp stages.

The most enriched gene in the medusa (supplementary Appendix H, Supplementary Material online), *ABCA3*, plays a role in the transmembrane transportation of surfactants. These substances reduce the surface tension of liquids and crucial for normal respiratory development and processes in vertebrates (Anandarajan et al. 2009). This may be because hydromedusae produce mucins, a natural polymeric surfactant (Uzawa et al. 2009; Petrou and Crouzier 2018) in their mesoglea, an extracellular matrix absent in polyps. Collagens, components of the mesoglea (Schmid et al. 1991), were also upregulated in the medusa (table 2). Similarly, collagen is enriched in the medusa stage in comparison to polyps in the hydrozoan *Podocoryna carnea* (Sanders and Cartwright 2015).

Consistent with the notion that medusae (but not polyps) contain smooth muscles and have a more complex sensory and nervous system, we find numerous genes that contribute to muscle development and contractions (table 2), and genes involved in neuron development and transmission (table 2 and  supplementary Appendix H, Supplementary Material online) upregulated in the medusa. Among those is dystroglycan, the major mediator of the integrity of muscles with some consistent functions from invertebrates to mammals (Shcherbata et al. 2007; Adams and Brancaccio 2015). Dystroglycan also functions as a critical regulator of proper development and of the nervous system in Metazoa (Yatsenko and Shcherbata 2014; Lindenmaier et al. 2019). On the other hand, we show an enrichment of genes involved in chitin metabolic processes and the formation of a primary germ layer in the polyps (supplementary Appendix I, Supplementary Material online), likely reflecting the polyps’ chitinous protective exterior and continuous allocation of energy toward asexual budding to expand the colony.

Overall, consistent with morphological and physiological differences between the medusa and polyp stages, our analyses identify DEGs associated to transmembrane transport, components of the mesoglea (e.g., mucin, collagen, etc.), muscular development and usage, neuronal development, and increased ability to respond to external stimuli in the medusa, and DEGs associated with chitin metabolism and formation of primary germ layer in the polyp. Furthermore, the top 50 DEGs in the medusa showed an unusually high number of novel transcripts. This may reflect the low number of published genomes of Hydrozoa with a medusa stage (the top BLASTx hit distribution shown in figure 1 includes Cnidaria without medusa) and the fact that, unlike other cnidarians, the medusae of *T. dohrnii* undergo reverse development. The study of Cnidaria with a variety of life cycles is needed to progress toward a better understanding of the genetics and evolution of their life stages.

## Conclusion

The transcriptome assembly and profiling presented in the paper revealed that the cyst stage of *T. dohrnii* is enriched with genes that are associated with aging/lifespan, regulation of transposable elements, DNA repair and damage response, and Ubiquitin-related processes. We show that a large portion (44%) of the top 50 DEGs in the cyst, a unique life cycle stage within the Hydrozoa, are novel and not annotated. We also show that polyps from the two different developmental trajectories (medusa reversal and budding within a wild colony) exhibit significant differences in gene activity, with processes of chromatin remodeling, matrix metalloproteinases, and embryonic development being highly active in the reversed polyp. The polyp and medusa also show major differences, with transmembrane transport, nervous system, components of the mesoglea, and muscle contraction-related categories being overexpressed in the medusa. In contrast, categories related to chitin metabolism and the formation of primary germ layers are overexpressed in the polyp. Ultimately, we produce genomic tools in the form of a high-quality transcriptome and provide insight into genes and genetic networks associated with each of its life cycle stages and with the reverse development of *T. dohrnii*.

## Materials and Methods

### Specimen Collection, Rearing, and Identification

A *T. dohrnii* colony bearing medusa buds was collected from Bocas del Toro, Panama in July 2015. Polyp hydranths were cut off the colony and preserved in RNAlater (ThermoFisher Scientific, Catalog #: AM7020). Released medusae were isolated into petri dishes, and a few of the medusa and the remaining colony was preserved. The remaining medusae were starved until they formed into cysts and subsequently reversed into the polyp stage. As there is variability in the time it takes for individual medusa to form into a cyst, the stage was preserved when the following physical attributes were visible (Schmich et al. 2007): 1) Cyst has no visible physical attributes remaining from the medusa (i.e. tentacles, mesoglea, etc.); 2) Settled and attached onto a substrate; 3) Cyst shows a smooth, yellow-orange perisarc (protective chitinous exterior); and 4) Cyst has not formed any stolon. Prior to RNA extraction, total DNA was extracted from spare tissue from the colony all the specimens originated from (Zietara et al. 2000). A fragment of the mitochondrial 16S gene was amplified (Miglietta et al. 2007) to confirm the species of the samples (Acc.#: MH029858, Miglietta et al. [2018]).

### RNA Extraction/Library Construction/Sequencing

Total RNA was extracted from biological triplicates of polyp hydranths, medusae, cysts, and reversed polyps using the Epicentre kit (MasterPureTM RNA Purification kit #MCR85102). The Poly A-based SMARTer Ultra Low Input RNA kit for Sequencing v4 kit (Clontech Laboratories) was utilized on all RNA samples (total of 12). The cDNA generated in the amplification was constructed into libraries and sequenced in a single lane of the Illumina HiSeq 4000 platform (Illumina Sequencing Technologies). In total, 150 bp paired-end reads were generated for each library. Only two of the three generated cyst RNA-seq replicate data sets were viable for further use, which is elaborated in detail below (described in detail in Matsumoto et al. [2019] and in supplementary Appendix A, Supplementary Material online). The remaining 11 RNA-seq data sets were utilized in our paper. The RNA-seq data sets from the two cyst replicates were utilized in preliminary analyses (Matsumoto et al. 2019) and can be found under GenBank accession #: SRR10053756 and SRR10053757 (Under BioProject: PRJNA563171, BioSample ID: SAMN1266994).

### De Novo Transcriptome Assembly

Specific parameters, databases, and command lines for bioinformatic software and algorithms can be found in detail in supplementary Appendix J, Supplementary Material online. All data sets of RNA-seq reads were mapped to the existing polyp and medusa *T. dohrnii* transcriptomes from the Mediterranean Sea (Matsumoto et al. 2019), and their % mapped, broken reads, and estimated paired distances were used to ensure proper and adequate sequencing quality. One of the cyst replicates had poor alignment rates against the polyp transcriptome, high percentage of broken reads, few PE reads mapped, and an abnormal range of estimated paired distance in comparison to the other cyst replicates and other sequenced stages (Matsumoto et al. 2019; supplementary Appendix A, Supplementary Material online). Thus, the replicate was removed from subsequent analyses and transcriptome assembly. The remaining data sets were trimmed, normalized, and assembled using Trinity (Haas et al. 2013).

### Completeness of Transcriptome

Results outputted from Trinity’s in silico normalization and all individual libraries were separately mapped back to the assembled transcriptome using two different stringencies in the CLC Genomic Workbench v8 alignment software (supplementary Appendix A, Supplementary Material online). BUSCO v2.0 (Simão et al. 2015) with the Metazoa database was used to evaluate the completeness of the transcriptome in terms of gene content and broadly assess transcript fragmentation.

### Functional Annotation of Transcriptome and Biological Contaminant Removal

The Kraken2 sequence classifier tool (Wood and Salzberg 2014) was used to filter contaminant sequences from Bacteria, Archaea, and Virus sources, and contigs less than 400 bp were removed from the transcriptome prior to annotation as they tend to provide less biological meaning than longer transcripts, commonly having poor coverage and quality, and often lack proper protein assignments and function (Kitchen et al. 2015; supplementary Appendix B, Supplementary Material online). Moreover, trimming reads less than 400 bp has been applied to other transcriptomes among Cnidaria (Kitchen et al. 2015; Sanders and Cartwright 2015).

The OmicsBox software’s (BioBam) annotation pipeline (Conesa et al. 2005; Götz et al. 2008) was used to assign functional terms to assembled contigs. BLASTx via CloudBlast (Matsunaga et al. 2008) was performed, and all contigs with hits were assigned names based on the best BLAST description annotator tool within OmicsBox. InterProScan (IPS) (Zdobnov and Apweiler 2001) was used to build upon and confirm existing GO annotations. The InterPro annotations were merged and ANNEX Augmentation and the manual removal of 1st level annotations were conducted as recommended (Götz et al. 2008). EggNOG was also utilized to map, confirm, and merge GO terms to the B2G and IPS annotations. KEGG enzyme GO-enzyme code mapping was additionally performed and visualized in Blast2GO. Contigs that were not annotated by BLASTx, IPS, EggNOG, or KEGG were further annotated using the RFAM database for noncoding RNA, and BLASTn against the hydrozoan EST database and subsequent BLASTx search.

Biological contaminants were inferred through the generated top BLASTx hits chart. Contigs that had the best BLASTx alignment scores (i.e., top hit) to the following genera, *Thecamonas*, *Acanthoamoeba*, *Planoprotostelium*, *Abelmoschus*, and *Acytostelium*, that showed higher than 95% sequence similarity were eliminated from the transcriptome. The remaining contigs that had the highest hit to one of the named genera were reblasted against the Metazoa (taxa ID: 33208) NR database. Contigs that had no metazoan hits were predicted as biological contaminants and removed from the transcriptome before further downstream analyses due to the concern of biological contamination from culturing/rearing and epibiontic relationships. The remaining contigs were used for further downstream analyses.

### DGE Analyses

Sequencing reads from each of the 11 libraries were individually mapped back to the transcriptome to generate count data for all samples. Gene-level estimation-based count data were generated using RSEM (Li and Dewey 2011). EdgeR (Robinson et al. 2010) was utilized to filter and normalize the count data, and the outputted count data were utilized to conduct gene-level pairwise DGE analyses on the following: 1) Colonial Polyp versus Reversed Polyp; 2) Medusa versus Polyp. Sequential DGE analyses were conducted using the maSigPro Bioconductor package (Nueda et al. 2014) on the stages in the following order: Colonial Polyp, Medusa, Cyst, and Reversed Polyp. Significant DE genes were clustered into nine different gene expression profiles (i.e. gene activity pattern—linear, curved, etc.) using hierarchical clustering. Visualized expression profiles of representative genes in figure 3 were chosen based on the highest DEGs from each category with the most consistent expression among the stage replicates from each category listed in table 1*A* (supplementary Appendix F, Supplementary Material online).

### Functional Gene Enrichment Analyses

As all transcript isoforms belonging to a single unigene are combined for gene-level analyses, the transcript/isoform over 800 bp with the most GO annotated terms was utilized as the representative for the gene-level functional enrichment analyses. If no transcripts were over 800 bp, the transcript with the most GO terms was used as the gene representative. Functional gene enrichment analyses were conducted on the DE genes using the FatiGO (Al-Shahrour et al. 2004). Two-tailed Fischer’s Exact Tests were performed on pairwise and sequential DGE analyses. The biological processes GO domain was the focal domain of interest for subsequent analyses.

## Supplementary Material

Supplementary data are available at *Genome Biology and Evolution* online.

## Supplementary Material

evab136_Supplementary_Data
