# Supplementary material for: Cellular Reprogramming and Immortality: Expression Profiling Reveals Putative Genes Involved in Turritopsis dohrnii’s Life Cycle Reversal
Source: Genome Biol Evol. 2021 Jun 16;13(7):evab136. doi: 10.1093/gbe/evab136 (PMC8480191; doi:10.1093/gbe/evab136)
Supplement: evab136_Supplementary_Data [file evab136_supplementary_data.zip › Appendix_ABCDE_MAY2021.pdf]

Appendix A: Specimen collection and identification, pre-assembly processing and post-assembly quality assessments

Specimen collection and identification

All *T. dohrnii* samples were collected in July 2015 in Bocas del Toro, Panama. To reduce the genetic and sampling variability that may occur among different *T. dohrnii* individuals (e.g. collection site/time, potential of different sex, etc.), all biological replicates of the different stages (polyp, medusa, cyst, reversed polyp) originated (i.e. cut, liberated or induced by rearing after starvation) from a single colony. The mitochondrial 16S region was sequenced from the colony, resulting in a 100% identical match (e-value: 0.0) with *T. dohrnii* from the Panamanian region from NCBI. The generated 16S region was deposited to GenBank under the accession number MH029858.

Transcriptome assembly

The quality of the RNA-seq reads from each library was assessed by mapping each of the datasets to the previously published *T. dohrnii* polyp and medusa transcriptomes from the Mediterranean Sea [1]. As discussed in Matsumoto et al. (2019) [1], the Cyst 2 dataset was excluded from the analyses due to the abnormal number of reads mapped to the polyp transcriptome (9.68%) and range of estimated paired distance values (0-3,830bp) in comparison to the other *T. dohrnii* libraries (Table A1). The remaining 11 *T. dohrnii* libraries were confirmed as viable for transcriptome assembly.

Table A1- Read mapping quality assessment of RNA-seq reads of each *T. dohrnii* library from the Bocas del Toro, Panama region using the CLC alignment algorithm. [* = from Matsumoto et al. (2019) [1]; [R] = dataset removed from further analyses]

	Reference: Polyps from Mediterranean Sea			Reference: Medusa from Mediterranean Sea		
**Library**	**Mapping %**	**Broken Read %**	**Est. Paired distance**	**Mapping %**	**Broken Read %**	**Est. Paired distance**
Cyst 1 *	92.98%	10.61%	128-594 bp	94.90%	14.36%	130-594 bp
Cyst 2 *[R]	9.68%	8.73%	0-3830 bp (few pairs mapped)	91.91%	60.54%	0-240 bp
Cyst 3 *	90.97%	14.65%	172-596 bp	88.71%	15.02%	128-596 bp
Medusa 1	91.08%	9.85%	128-618 bp	94.37%	14.30%	128-620 bp
Medusa 2	91.98%	17.19%	212-487 bp	94.45%	11.31%	128-606 bp
Medusa 3	91.32%	17.67%	223-498 bp	94.05%	12.35%	128-596 bp
Polyp 1	93.14%	15.44%	205-479 bp	91.74%	10.26%	128-582 bp
Polyp 2	93.51%	19.72%	221-510 bp	91.58%	14.55%	128-606 bp
Polyp 3	90.95%	14.31%	128-598 bp	87.00%	17.93%	128-602 bp
Reversed Polyp 1	90.39%	31.65%	178-586 bp	87.32%	31.86%	128-580 bp
Reversed Polyp 2	83.74%	34.15%	126-534 bp	82.42%	36.70%	114-573 bp
Reversed Polyp 3	78.45%	44.63%	117-603 bp	75.33%	43.68%	108-636 bp

The datasets from the eleven RNA-seq libraries (Table A2) were pooled and were fed into the Trinity software [2] totaling in ~270 million paired-end (~ 540 million total) input reads (Table A3; see Appendix L for detailed method parameters). The reads were trimmed based on quality using Trimmomatic and the redundancy was reduced via in silico normalization within the Trinity package, resulting in total of ~13.4 million (~26.8 million) reads for assembly.

Table A2. Total number of reads for each generated *T. dohrnii* library.

Library	Total Reads	Paired
Cyst 1 *	51,524,006	25,762,003
Cyst 3 *	38,495,812	19,247,906
Medusa 1	54,698,848	27,349,424
Medusa 2	48,713,252	24,356,626
Medusa 3	58,698,848	29,349,424
Polyp 1	40,201,176	20,100,588
Polyp 2	49,104,256	24,552,128
Polyp 3	47,558,790	23,779,395
Reversed Polyp 1	51,715,980	25,857,990
Reversed Polyp 2	46,315,858	23,157,929
Reversed Polyp 3	51,298,702	25,649,351

Table A3. Total number of raw, trimmed and normalized reads from the pooled *T. dohrnii* dataset.

	Total Reads	Paired
Raw Reads	538,159,214	269,079,607
After trimming	537,256,754	268,628,377
After normalization	26,804,068	13,402,034

The first and foundational transcriptome assembly (~323.124 Mbp) resulted in a total of 416,169 transcripts, 317,473 unigenes with a GC content of 38.86% and a maximum contig

length of 27,093bp. The N50 of the entire assembly was 1,329bp with a median contig length of 408bp, and an average length of 776.43bp. Based on the longest unigenes (i.e. trinity 'genes/unique transcripts'), the assembly was ~184.450 Mbp with a N50 of 757bp, median contig length of 350bp, average length of 580.99bp.

Transcriptome Quality Assessments

The outputted reads from the *in-silico normalization* within trinity were mapped back to the assembled contigs to assess the completeness of the transcriptome in respect of the sequencing reads. The following stringency parameters were utilized with the CLC Genomic Workbench v8 alignment tool in the analyses: Medium- Length fraction=0.5, Similarity fraction=0.8; Stringent= Length fraction=0.8, Similarity fraction=0.8). A high number of the reads were mapped in both analyses, 95.6% and 92.4%, respectively, indicating that very little information was lost in the unassembled reads (Table A4).

Table A4. Read mapping analyses of transcriptome using the CLC Genomic Workbench alignment tool. [LF= length fraction; SF= similarity fraction; Estimated paired distance of the reads: 128-610 bp]

	**Medium (LF=0.5, SF=0.8)**	**Stringent (LF=0.8, SF=0.8)**
Input Reads (normalized)	26,804,068	
Mapped Reads	25,614,004	25,757,822
Read Mapping %	95.6%	92.4%

To ensure that all libraries in each stage were well represented in the assembled transcriptome, the raw reads were individually mapped back to the assembled transcripts (Table A5). All libraries within each stage were highly represented in the transcriptome, in which the mapped read percentage ranged from 97.63-99.41% using stringent parameters. Overall, a very high percentage of the sequencing reads were incorporated into our assembled transcriptome, indicating a highly complete assembly.

Table A5: Raw paired-end reads from each library mapped back to assembled transcriptome.

Library	Mapped Reads	Mapping %
Cyst 1	51,218,630	99.41%
Cyst 2	38,196,626	99.22%
Polyp 1	39,941,300	99.35%
Polyp 2	48,645,838	99.07%
Polyp 3	46,973,254	98.77%
Medusa 1	54,243,416	99.17%
Medusa 2	48,343,380	99.25%
Medusa 3	58,129,597	99.31%
Reversed Polyp 1	51,066,990	98.75%
Reversed Polyp 2	45,218,859	97.63%
Reversed Polyp 3	50,176,926	97.81%

The BUSCO [3] tool was utilized to determine the completeness of the transcriptome in terms of gene content using the Metazoa database. The Metazoa analysis reported 97.9% completeness (95.6% complete, 2.2% partial), indicating that our assembly is highly complete in terms of gene content (Table A6).

Table A6: Gene content completeness analyses of the transcriptome using the BUSCO tool.

	**Metazoa database** Total BUSCOs: 978
**Complete BUSCOs**	935 (95.6%)
**Partial BUSCOs**	22 (2.2%)
**Complete+Partial BUSCOs**	957 (97.9%)
**Missing BUSCOs**	21 (2.2%)

References

1. Matsumoto Y, Piraino S, Miglietta MP: **Transcriptome characterization of reverse development in Turritopsis dohrnii (Hydrozoa, Cnidaria)**. *G3: Genes, Genomes, Genetics* 2019, **9**(12):4127-4138.
2. Haas BJ, Delcher AL, Mount SM, Wortman JR, Smith Jr RK, Hannick LI, Maiti R, Ronning CM, Rusch DB, Town CD: **Improving the Arabidopsis genome annotation using maximal transcript alignment assemblies**. *Nucleic acids research* 2003, **31**(19):5654-5666.
3. Simão FA, Waterhouse RM, Ioannidis P, Kriventseva EV, Zdobnov EM: **BUSCO: assessing genome assembly and annotation completeness with single-copy orthologs**. *Bioinformatics* 2015, **31**(19):3210-3212.

Appendix B: Transcriptome trimming and filtering of biological contaminants

Bacteria, Archaea and Viral contaminant removal

Though sequences Poly A-tails were excluded during cDNA library preparation to exclude the majority of bacterial sequences, the Kraken metagenomic classification tool [1] was utilized to further filter contigs based on operational taxonomic units (OTUs) that belong to Bacteria, Archaea and virus species (database: All Bacterial, Archaeal and Viral Genomes in RefSeq). Out of the 416,169 assembled contigs, 9,616 sequences (2.31%) were classified to be from bacterial, archaeal and viral sources (Figure B1). Despite excluding prokaryotic sequences from our cDNA library, the metagenomic classification can be useful to provide insight on the bacterial, archaeal and viral species that are part of the microbiome within *T. dohrnii* and/or of the environment in Bocas del Toro, Panama (Atlantic). The most common contaminant taxonomic class among Bacteria, Archaea and Viral groups was Gammaproteobacteria, representing 56% of the contaminant classified contigs (Figure B1). Other common classes include Actinobacteria (21%), Alphaproteobacteria (18%) and Bacilli (18%). The new transcriptome statistics with classified sequences removed is reported in Table B1 (filtered contaminants #1).

Superkingdom

AllTurriPanama		
Taxa	Count	%
Bacteria <prokaryotes>	8583	2.06
Viruses	601	0.14
Archaea	432	0.1

Phylum

AllTurriPanama		
Taxa	Count	%
Proteobacteria	4352	1.04
Firmicutes	1111	0.26
Bacteroidetes <phylum>	965	0.23
Actinobacteria <phylum>	901	0.21
Euryarchaeota	398	0.09
Tenericutes	226	0.05
Cyanobacteria	180	0.04
Spirochaetes <phylum>	59	0.01
Planctomycetes <phylum>	33	0.0
Crenarchaeota <phylum>	20	0.0

Class

AllTurriPanama		
Taxa	Count	%
Gammaproteobacteria	2336	0.56
Actinobacteria <class>	879	0.21
Alphaproteobacteria	758	0.18
Bacilli	756	0.18
Flavobacteriia	664	0.15
Betaproteobacteria	528	0.12
Clostridia	295	0.07
Epsilonproteobacteria	278	0.06
Deltaproteobacteria	246	0.05
Mollicutes	222	0.05

Order

AllTurriPanama		
Taxa	Count	%
Flavobacteriales	664	0.15
Bacillales	643	0.15
Alteromonadales	523	0.12
Burkholderiales	403	0.09
Enterobacterales	347	0.08
Pseudomonadales	308	0.07
Oceanospirillales	302	0.07
Campylobacterales	276	0.06
Clostridiales	256	0.06
Rhizobiales	221	0.05

Family

AllTurriPanama		
Taxa	Count	%
Flavobacteriaceae	566	0.13
Bacillaceae	434	0.1
Alteromonadaceae	373	0.08
Campylobacteraceae	245	0.05
Pseudomonadaceae	236	0.05
Streptomycetaceae	211	0.05
Rhodobacteraceae	197	0.04
Burkholderiaceae	188	0.04
Clostridiaceae	181	0.04
Mycoplasmataceae	173	0.04

Genus

AllTurriPanama		
Taxa	Count	%
Bacillus <bacterium>	411	0.09
Alteromonas	282	0.06
Pseudomonas	232	0.05
Arcobacter	206	0.04
Streptomyces	200	0.04
Clostridium	177	0.04
Mycoplasma	173	0.04
Methanosarcina	151	0.03
Staphylococcus	150	0.03
Vibrio	130	0.03

Species

AllTurriPanama		
Taxa	Count	%
Bacillus cereus	205	0.04
Alteromonas macleodii	162	0.03
Clostridium botulinum	111	0.02
Mycoplasma hyopneumoniae	101	0.02
Cutibacterium acnes	86	0.02
Pandoravirus salinus	72	0.01
Winogradskyella sp. J14-2	61	0.01
Arcobacter sp. LPB0137	58	0.01
Methanococcus voltae	57	0.01
Staphylococcus cohnii	56	0.01

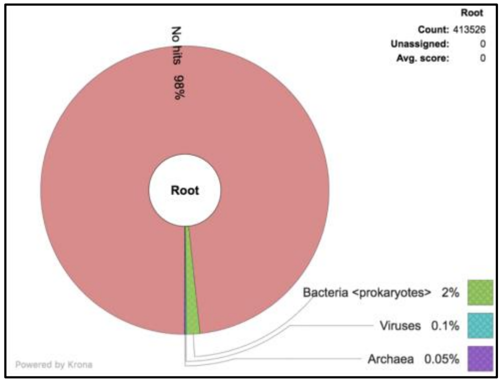

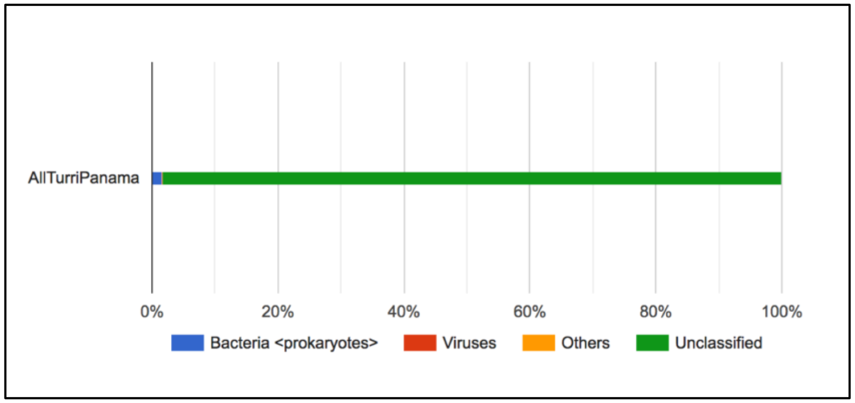

Contaminant Taxonomic Class

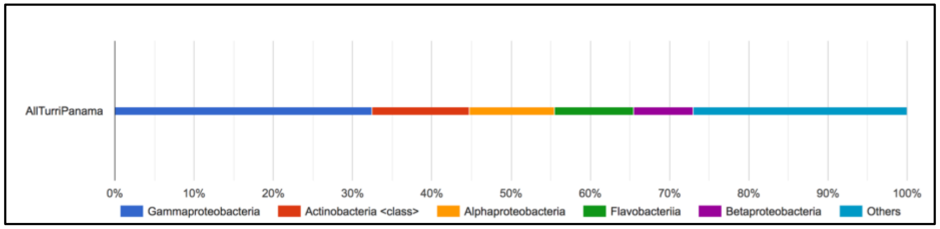

Figure B1: Classified contigs from Bacteria, Archaea and Viral sources (top 10 taxa from each group). [Database: All RefSeq Bacteria, Archaea and Virus genomes].

Only contigs that were larger than 400bp were kept for further analyses. This approach is similar to that applied to other Cnidarian transcriptomes [2-4]. Six duplicated sequences (i.e. exactly the same) were removed from the transcript dataset. Short contigs are less likely to provide biological meaning to our analyses as they tend to have poor coverage and quality (e.g. artifacts), often under-represented, often have no assigned protein or function (i.e. functional annotation) and can come from contaminant, non-target organisms. Though they may provide some information in RNA-seq analyses, short sequences are harder to validate with complications in statistical power, confidence and biological interpretation as mRNA transcripts are longer in natural systems. In the *T. dohrnii* cyst transcriptome that was annotated in Matsumoto et al. (2019) [5], only 14.40% of the contigs shorter than 400 bp had blast hits and 9.61% were annotated with GO terms.

Our newly trimmed transcriptome (~258.305 Mbp) resulted in 206,159 transcripts and 129,607 unigenes with a GC content of s38.38% (Table B1). The new N50 of the trimmed assembly is 1,725 with a median contig length of 828 bp and an average length of 1,252.91 bp. Based on the longest unigenes (~128.976 Mbp), the N50 was 1,185 bp with a median length of 676 bp and average length of 995.11 bp.

Table B1. Assembly statistics for the transcriptomes for the pooled *T. dohrnii* dataset. [Pre-trim/filter=original assembly; Filtered contaminants= Bacteria, Archaea and Viral species filtered using Kraken metagenomic classifier; Trimmed= only contigs larger than 400bp kept; Filtered contaminants #2 = biological contaminants found in annotations filtered from dataset (see methods)]

		Pre-trim/filter	Filtered contaminants #1	Trimmed (>400bp)	Filtered contaminants #2
	**# of unique transcripts**	317,473	310,142	129,603	127,645
	**# of transcripts**	416,169	406,553	206,159	204,031
	**% GC**	38.86%	38.72%	38.38%	38.29%
	**Minimum length**	201	201	401	401
	**Maximum length**	27,093	27,093	27,093	27,093
Based on all transcripts	**Contig N10**	4,268	4,259	4,584	4,595
	**Contig N20**	3,076	3,069	3,415	3,426
	**Contig N30**	2,347	2,342	2,696	2,706
	**Contig N40**	1,785	1,780	2,163	2,173
	**Contig N50**	1,329	1,324	1,725	1,734
	**Median contig length**	408	406	828	832
	**Mean contig length**	776.43	773.35	1,252.92	1258.07
	**Total assembled bases**	~323.124 Mb	~314.406 Mb	~258.301 Mb	~256.685
Based on longest unigene	**Contig N10**	3,348	3,338	3,909	3,928
	**Contig N20**	2,147	2,139	2,706	2,724
	**Contig N30**	1,472	1,462	2,013	2,028
	**Contig N40**	1,044	1,034	1,627	1,538
	**Contig N50**	757	749	1,185	1,194
	**Median contig length**	350	349	675	676
	**Mean contig length**	580.99	577.9	995.12	998.99
	**Total assembled bases**	~184.450 Mb	~179.231 Mb	~128.971 Mb	~127.517

BLASTx Eukaryota biological contamination removal

BLASTx using the NCBI's Non-Redundant (NR) database was performed on the filtered, deduplicated and trimmed contigs (206,159 transcripts) using a e-value cutoff of e^{-3}. The species distribution of all BLAST hits (maximum number of hits per contig: 20) indicated that the top three most represented taxa are *Stylophora pistillata* (120,307 hits), *Hydra vulgaris* (104,252 hits) and *Exaiptasia pallida* (101,621 hits) (Figure B2), and the overall top six taxa were all cnidarians (indicated in green). There are, however, three taxa that could represent biological contaminants, *Acanthamoeba, Thecamonas,* and *Acytostelium* species (indicated in red).

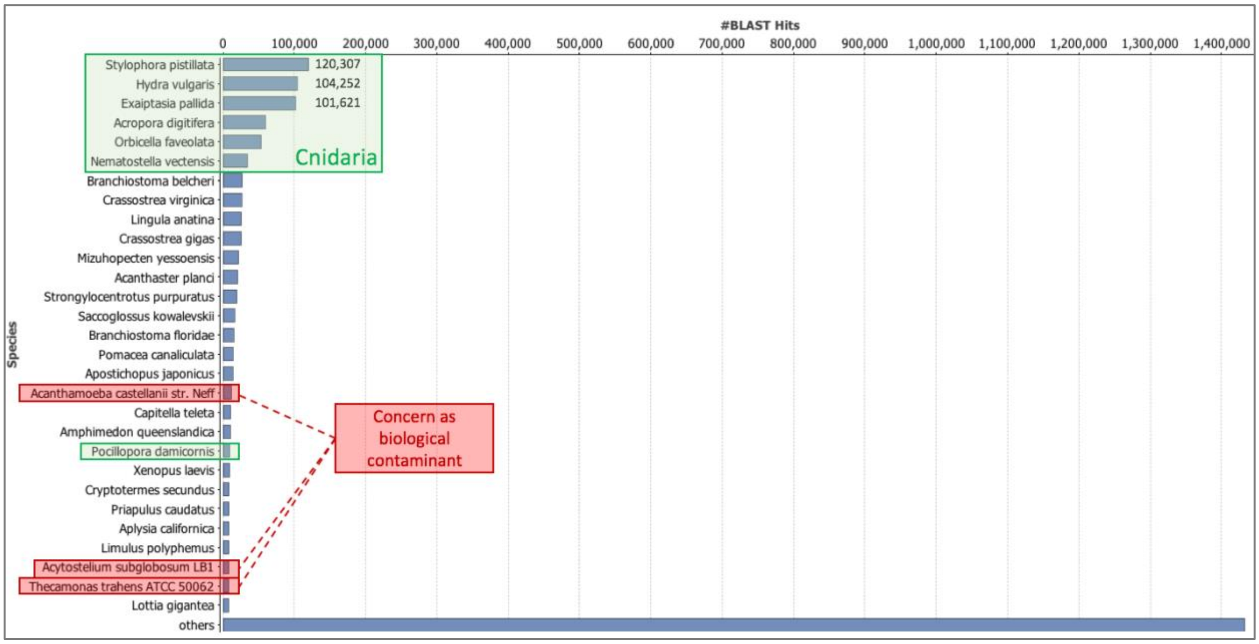

Figure B2: Species distribution of all BLASTx hits (pre-filter biological contaminant #2). [total contigs with hits: 108,060; top 20 blast hits saved; Green: Cnidarian taxa; Red: Taxa concerned as biological contaminant]

A closer look at the top-hit species distribution shows that there is an unusually high representation of fungal and protozoan species (e.g. slime molds, amoebas) (indicated in red), following or ranked between cnidarian taxa (indicated in green) (Figure B3). A total of 8,564 contigs had top-hits that belonged to the following five genera of concern: *Thecamonas*, *Acanthamoeba*, *Planoprotostelium*, *Abelmoschus* and *Acytostelium*. The sequence similarity distribution of the predicted contaminant sequences indicates a number of sequences that are highly similar (>95% sequence similarity indicated in red box, Figure B4) to the named taxa (565 sequences with top hits greater than 95%), indicating towards true biological contaminants. These sequences were removed from our transcriptome before subsequent analyses.

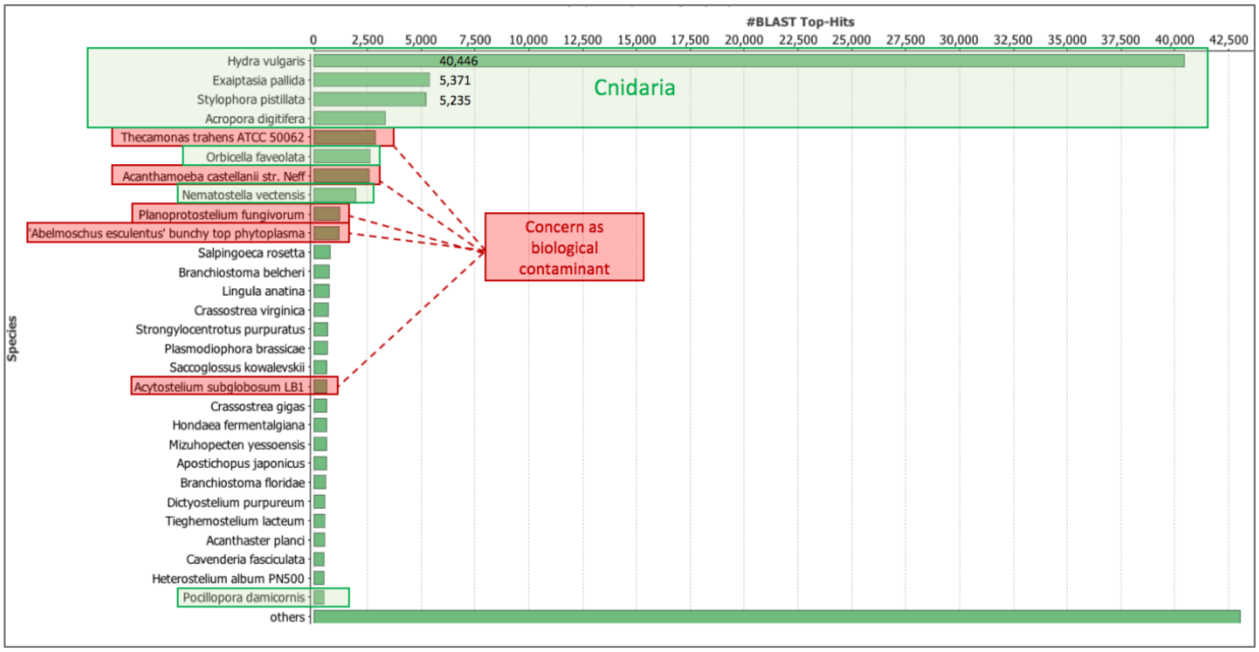

Figure B3: Species distribution of top BLASTx hits (pre-filter biological contaminant #2). [total contigs: 108,060; Green: Cnidarian taxa; Red: Taxa concerned as biological contaminant]

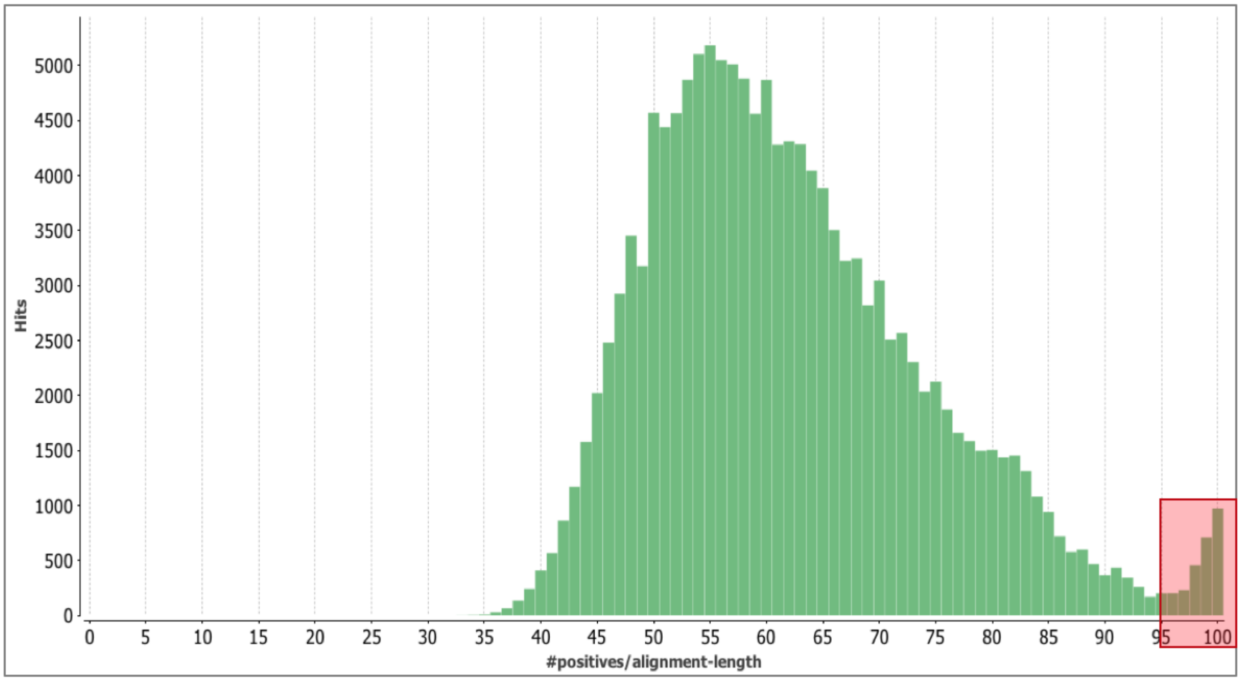

Figure B4: Sequence similarity distribution of contaminant BLASTx hits. [Genera: *Thecamonas, Acanthamoeba*, *Planoprotostelium*, *Abelmoschus* and *Acytostelium*]

Additionally, the species distribution of the hits among predicted contaminant sequences indicate that a large number of the hits belong to non-metazoan taxa, particularly fungal and protozoan, with exception of a number of metazoans (indicated in orange) (Figure B5). The

contigs that incorporate metazoan hits are likely from *T. dohrnii* and not a contaminant. Among the metazoan taxa were cnidarians (*S. pistillata*, *E. pallida*), lancelets (*B. belcheri*) and mollusks (*L. anatina, Crassostrea* sp. and *P. canaliculata*). Due to the nature of the laboratory rearing of the cyst and reversed polyp stages, and the marine benthic environment of polyp colonies, fungal and epibiontic organisms (living associated with various taxa) are probable to be present as biological contaminants within our transcriptome. Figure B6 portrays *T. dohrnii* polyps that were growing on a crab with other epibiontic species in the shallow and tropical waters of Bocas del Toro, Panama (Atlantic). Additionally, Matsumoto et al., (2019) [5] reported contamination of the foraminiferan *Reticulomyxa filosa* (Protist) in the individually constructed polyp transcriptome of *T. dohrnii* from the Mediterranean Sea, Italy. Epibiontic protists have been found on hydrozoan colonies [6]. To best avoid contamination from such organisms, precautions were taken during specimen collection, where individual polyp hydranths from the top of the colony with the least amount of visible fouling organisms were cut off and preserved for subsequent processing. In our newly assembled transcriptome from Bocas del Toro, Panama, there was a total of 300 sequences with top-hits that belong to the genus *Reticulomyxa* and only 2 had >95% sequence similarity with both sequencing incorporating Metazoa among the top 20 hits, confirming that there is very little concern for *R. filosa* contamination in the polyp sequencing reads.

To best ensure that the sequences belonged to actual biological contaminants, the contigs were re-blasted against the NR Metazoa database (taxid: 33208) during subsequent IPS, EggNOG and KEGG annotation analyses. 1,563 out of 7,999 contigs that had no hits against metazoan proteins were predicted to be contaminant sequences from fungal or protozoan

sources, and thus removed from the transcriptome. In total, 2,128 contigs were removed from the transcriptome and a total of 204,031 contigs remain for subsequent annotation processing.

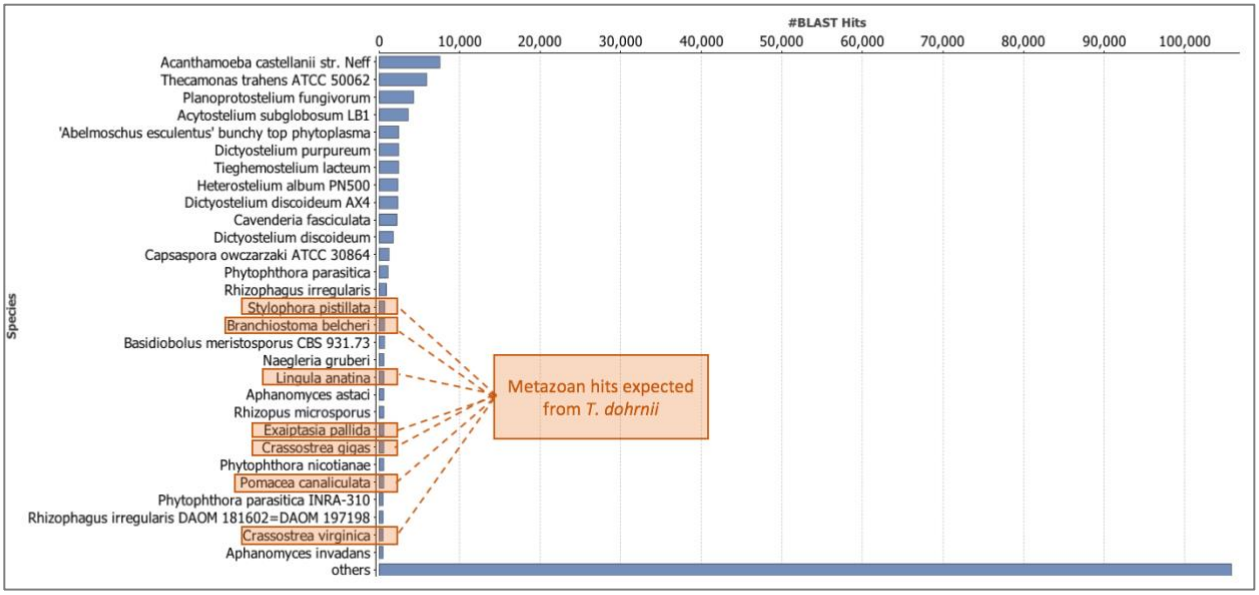

Figure B5: Species distribution of contaminant BLASTx hits. [Genera: *Thecamonas, Acanthamoeba, Planoprotostelium, Abelmoschus* and *Acytostelium;* Orange=Metazoan species]

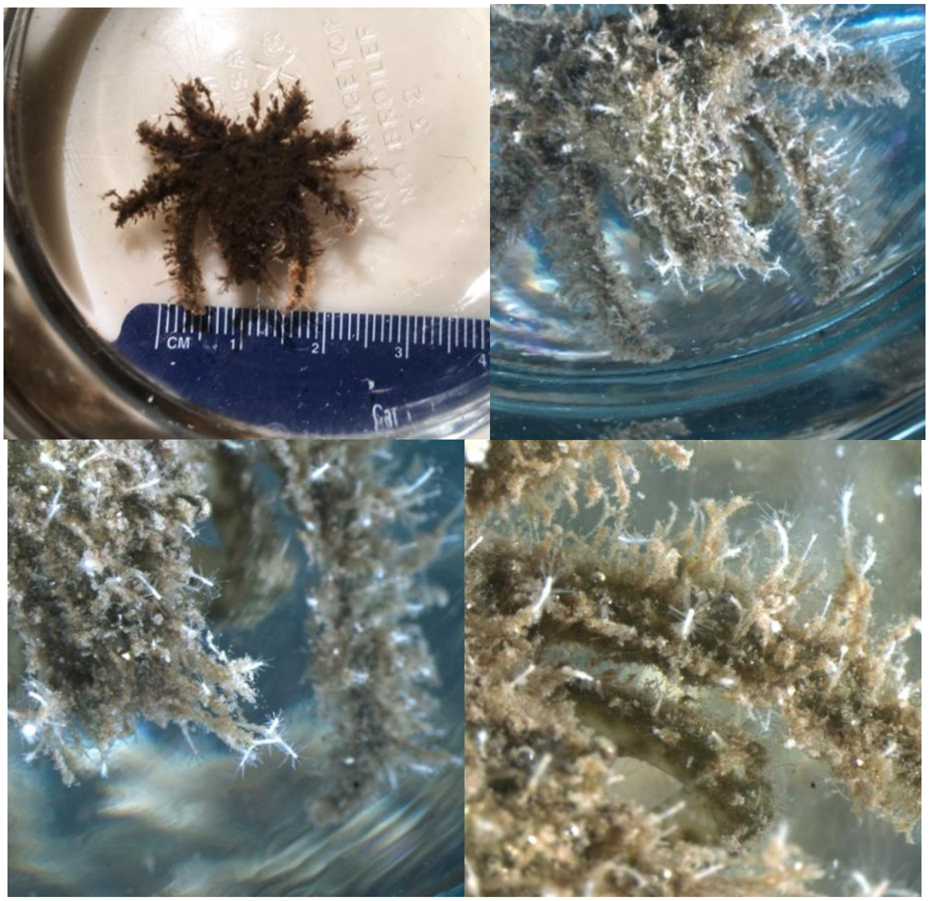

Figure B6: *T. dohrnii* polyps growing on the surface of a crab along with other epibiontic species in Bocas del Toro, Panama (Atlantic). [Top left: Entire crab; top right: closeup of crab anterior with polyps; bottom left: closeup of the oral region of the crab with polyps; bottom right: closeup of appendages of the crab with polyps].

The newly filtered transcriptome (~265.685 Mbp) resulted in 204,031 transcripts and 127,645 unigenes with a GC content of 38.29% (Table 4). The new N50 of the trimmed assembly is 1,734 bp with a median contig length of 832 bp and an average length of 1,258.07 bp. Based on the longest unigenes (~127.517 Mbp), the N50 was 1,194 with a median length of 676 bp and average length of 998.99 bp.

References

1. Wood DE, Salzberg SL: **Kraken: ultrafast metagenomic sequence classification using exact alignments**. *Genome biology* 2014, **15**(3):R46.
2. Kitchen SA, Crowder CM, Poole AZ, Weis VM, Meyer E: **De novo assembly and characterization of four anthozoan (phylum Cnidaria) transcriptomes**. *G3: Genes, genomes, genetics* 2015, **5**(11):2441-2452.

3. Sanders SM, Cartwright P: **Interspecific differential expression analysis of RNA-Seq data yields insight into life cycle variation in hydractiniid hydrozoans**. *Genome biology and evolution* 2015, **7**(8):2417-2431.
4. Sanders SM, Cartwright P: **Patterns of Wnt signaling in the life cycle of Podocoryna carnea and its implications for medusae evolution in Hydrozoa (Cnidaria)**. *Evolution & development* 2015, **17**(6):325-336.
5. Matsumoto Y, Piraino S, Miglietta MP: **Transcriptome characterization of reverse development in Turritopsis dohrnii (Hydrozoa, Cnidaria)**. *G3: Genes, Genomes, Genetics* 2019, **9**(12):4127-4138.
6. Bavestrello G, Cerrano C, Di Camillo C, Puce S, Romagnoli T, Tazioli S, Totti C: **The ecology of protists epibiontic on marine hydroids**. *Journal of the marine Biological Association of the United Kingdom* 2008, **88**(8):1611-1617.

Appendix C: Transcriptome functional annotation

BLAST2GO pipeline (BLASTx, GO mapping, annotation)

After contaminant removal, 115,932 out of 204,031 (56.82%) contigs had BLAST hits (Table C1). Among the hits, 96,438 contigs (83.18% out of sequences with BLAST hits) were mapped with GO terms, and 72,167 contigs (74.83% out of sequences with GO terms) were B2G annotated.

Table C1: Total number of annotated contigs based on each annotation method. [Total number of contigs in transcriptome: 204,031; Blue: Cumulative total number of contigs with any type of annotation; Green: Cumulative number of contigs B2G annotated with GO term, *- not included as annotation/gene description]

Total contigs in transcriptome: 204,031		
B2G	Contigs with Blast hits	115,932
	Contigs mapped with GO terms	96,438
	Contigs B2G annotated	72,167
	Total contigs with annotation	115,932
	Total contigs with GO term	72,167
IPS	Contigs with IPS hits	149,259
	IPS hits with GO term	61,642
	Total contigs with annotation	161,717
	Total contigs with GO term	78,162
KEGG	Contigs with KEGG hits w/GO term	11,005
	Total contigs with annotation	161,717
	Total contigs with GO term	78,162
COG	Contigs with COG hits w/GO term	67,514
	Total contigs with annotation	161,717
	Total contigs with GO term	85,782
Rfam	Contigs with Rfam hits	115
	Total contigs with annotation	161,832
	Total contigs with GO term	85,897
EST	Contigs with EST hits*	418
	EST hits with Blast hits	178
	Contigs mapped with GO terms	63
	Total contigs with annotation	162,010
	Total contigs with GO term	85,960

InterProScan annotations

Out of 204,031 total contigs, 149,259 sequences (73.16%) had at least one IPS hit and 61,642 sequences were annotated with GO terms (Table C1). The results from IPS were merged with the B2G annotations to confirm previous and find new GO terms. 67,362 new GO terms were added, totaling in 293,026 GO terms found in our transcriptome. 5,995 uncharacterized

sequences were newly were annotated with GO terms, and ultimately, a total of 78,162 contigs were annotated with a GO term.

KEGG annotations

The Kyoto Encyclopedia of Genes and Genomes (KEGG) database was utilized to map enzyme codes (EC) to our transcriptome. A total of 19,474 contigs were annotated with an EC (Figure C1). Among the EC classes, hydrolases were the most abundantly present, with 11,005 annotated contigs, then transferases with 4,881 annotated contigs, and oxidoreductases with 2,379 annotated contigs.

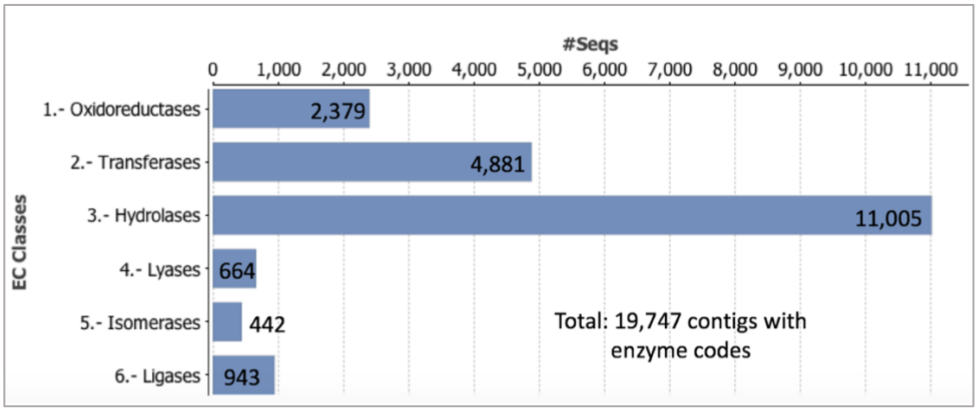

Figure C1: KEGG Enzyme code annotation distribution.

EggNOG annotations

The Conserved Orthologous Groups (COG) mapping tool against the EggNOG database within B2G was utilized to further annotate our transcriptomes with GO terms. A e-value cutoff of e^{-3} and bit score cutoff of 60 was used to only merge high quality annotations from EggNOG with our B2G and IPS annotations to confirm previous and find new GO terms. 67,514 sequences out of 204,031 total contigs (33.09%) were mapped with 1,031,536 GO terms found in the EggNOG database, and merged onto the existing B2G and IPS annotations to confirm and find new annotations. 11,551 contigs with no previous GO annotation were mapped, accumulating to 85,782 contigs mapped with at least one GO term.

RFAM annotations

Non-coding RNA (ncRNA) are mRNA sequences that do not get translated into a protein sequence, but are captured during RNA-seq as they have undergone transcription. ncRNA, also referred to previously as 'junk DNA', are common in animal genomes, but functions are not always known (Cheng et al., 2005; Birney et al., 2007; Bakel et al., 2010). Recent genomic and transcriptomic analyses have uncovered that they contribute to a number of human diseases, such as cancer and neurodegeneration (Esteller, 2011; Adams, 2017; Distefano, 2018; Lekka, 2018). The ncRNA database in Rfam was used to further characterized transcripts that had no prior annotation (i.e. no B2G, IPS, KEGG, EggNog, or hydrozoan EST annotations). A total of 42,251 contigs were analyzed. There were two uncharacterized contigs larger than 10,000 bp which was above the maximum input sequence length and could not be analyzed in Rfam. 169 new GO terms among 115 newly annotated contigs were merged to existing annotations. Among the annotated contigs, (Figure C2). Ultimately, 161,832 out of 204,031 contigs (79.32%) had at least one type of annotation (i.e. BLASTx, IPS, EggNOG, KEGG, Rfam) and 85,897 (41.10%) were annotated with GO terms.

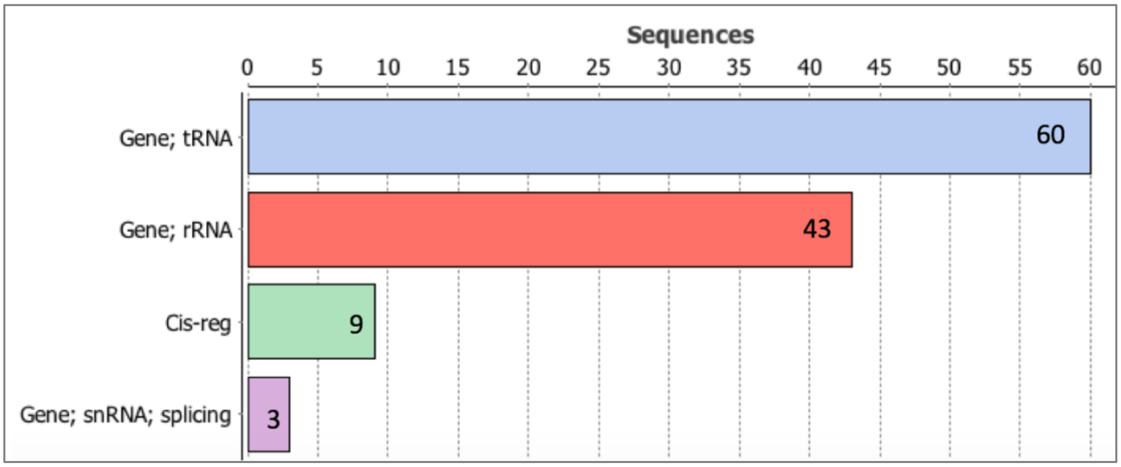

Figure C2: Rfam annotation sequence distribution where 169 new GO terms were added to 115 non-coding RNA sequences.

Hydrozoan EST/BLASTx annotations

RNA-seq data that are shorter in sequence length or constructed from lower quality reads can result in difficulties in finding the correct protein alignment. The uncharacterized sequences can first be aligned to the expressed sequence tag (EST) database, where it can then once again be compared to a protein databased to further increase the completeness of out transcriptome annotations. BLASTn using the Hydrozoa EST database (taxid: 6074, hydrozoans) was performed on transcripts that had no form of annotation (i.e. B2G, InterProScan, RFam, KEGG, EggNOG). Out of the 42,199 un-annotated transcripts, 418 transcripts had significant matches to hydrozoan EST sequences. These sequences were then compared to the Hydrozoa protein subset of the NR database (TaxID: 6074, hydrozoans) via BLASTx, and GO mapping/annotation was subsequently performed. 178 new contigs had Blast hits and 63 sequences were functionally annotated with GO terms. In total, 85,960 out of 204,031 contigs (42.13%) were annotated with GO terms and 162,010 contigs (79.40%) with any type of annotation from the utilized databases, specifically BLASTx, IPS, EggNOG, KEGG, Rfam and the Hydrozoa EST. There were ultimately 7,470 contigs with no annotation in the transcriptome that have potential to be novel transcripts from genes that have yet to be characterized. There were 7,470 total contigs over 1000 bp that did not have any annotation, particularly likely to be novel.

Appendix D: Pre-processing reads and alignment of RNA-seq libraries

Quality trimming and alignment of RNA-seq libraries

Illumina-based platforms has been reported to be prone to the decrease of sequencing quality towards the 3' end, subsequently negatively impacting read-mapping analyses [1]. However, for differential gene expression pipelines, aggressive trimming of sequencing reads is unnecessary and often detrimental for downstream analyses [2]. Prior to generating count tables for each individual library, reads were lightly trimmed based on quality from the 3' end with a phred score cutoff of 10, as recommended in [2]. Though the alignment generated both gene and transcript/isoform-based expression data, analyses were performed based only on gene level-based analyses (i.e. all transcripts/isoforms are categorized with the same gene and function; based only 'unique' trinity genes). Differentiating among different outcomes of alternative splicing (i.e. based on isoform/transcript-level analyses) is both interesting and important, but with no available genome for *T. dohrnii*, distinguishing between true isoforms and fragmentation of contigs is difficult and will result in erroneous expression data. This nature of transcriptome assemblies in contrast to genome assemblies is portrayed when comparing the transcriptome and genome of the same species [3, 4], where the number of total contigs in genomes are much higher than the number of total transcripts assembled. Using the assumption that most isoforms for a single gene will have similar GO terms and annotations, the longest isoform for each gene was used to represent GO annotations for each gene.

All libraries were normalized to eliminate systematic effects (i.e. differences in library size) and make accurate comparisons among stages and their replicates, and genes with low counts were filtered across libraries using a count per million (CPM) filter value of 1 (corresponding to approximate counts of 10-15 per gene) as recommended for DGE analyses [5].

Subsequently, the maSigPro Bioconductor package [6] for time-series DGE analyses was utilized to perform sequential DGE analyses in the following order of lifecycle stages: 1) Polyp (hydranth from colony), 2) Medusa, 3) Cyst, 4) Reversed Polyp. Differentially expressed genes were identified and categorize their gene-expression profiles (i.e. different models/patterns of gene activity) based on hierarchal clustering.

A multidimensional scaling plot (MDS) plot representing the differences in expression data between replicates and among stages was produced to confirm sample quality (Figure D1). A well-controlled experiment will portray the largest sources of expression variation to be between different lifecycle stages of replicates rather than among replicates

The MSD plot for our data portrays that the biological replicates for each stage cluster together in close proximity, particularly the Medusa stage (Figure D1, red) and the colonial Polyp stage (green), while the Cyst (purple) and the Reversed Polyp stage (blue) show the replicates being slightly more dissimilar. This could potentially be explained by the nature of the both of the stages collected. The cyst stage is the intermediate stage during the polyp-to-medusa rejuvenation, and thus, is more difficult to be consistent despite preservation during a specific time-point in which morphological traits appear or disappear (i.e. stage defined as attached to a surface with a complete perisarc). The reversed polyp is also a stage in which consistency is slightly more difficult to obtain for similar reasons, where each individual may be at a slightly different stage of the rejuvenation (i.e. just rejuvenated vs. moved onto elongating stolons to produce more polyps) despite the best attempt to preserve each replicate with exactly the same features (i.e. stage defined as the production of a single functional polyp). Additionally, the cyst and particularly the reversed polyp stages likely incorporate more biological contaminants from

culturing specimen in petri dishes, such as various fungi species reported in the pre-filtered BLAST results making samples less similar than the colonial polyp and medusa stage.

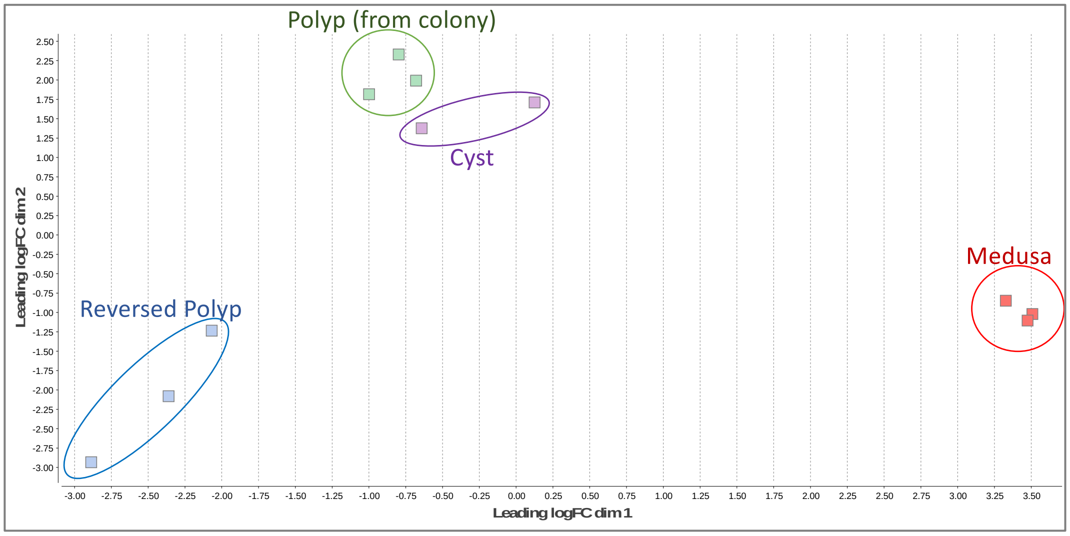

Figure D1: MDS plot of expression data of all eleven libraries used in the DGE analysis [Polyp (colony)-green, Medusa-red, Cyst-purple, Reversed Polyp-blue; LogFC- measure in change of expression, where + means upregulated and – means downregulated].

<u>References</u>:

1. Fuller CW, Middendorf LR, Benner SA, Church GM, Harris T, Huang X, Jovanovich SB, Nelson JR, Schloss JA, Schwartz DC: **The challenges of sequencing by synthesis**. *Nature biotechnology* 2009, **27**(11):1013.
2. Williams CR, Baccarella A, Parrish JZ, Kim CC: **Trimming of sequence reads alters RNA-Seq gene expression estimates**. *BMC bioinformatics* 2016, **17**(1):103.
3. Gold DA, Katsuki T, Li Y, Yan X, Regulski M, Ibberson D, Holstein T, Steele RE, Jacobs DK, Greenspan RJ: **The genome of the jellyfish Aurelia and the evolution of animal complexity**. *Nature ecology & evolution* 2019, **3**(1):96.
4. Brekhman V, Malik A, Haas B, Sher N, Lotan T: **Transcriptome profiling of the dynamic life cycle of the scypohozoan jellyfish Aurelia aurita**. *BMC genomics* 2015, **16**(1):74.
5. Chen Y, Lun AT, Smyth GK: **From reads to genes to pathways: differential expression analysis of RNA-Seq experiments using Rsubread and the edgeR quasi-likelihood pipeline**. *F1000Research* 2016, **5**.
6. Nueda MJ, Tarazona S, Conesa A: **Next maSigPro: updating maSigPro bioconductor package for RNA-seq time series**. *Bioinformatics* 2014, **30**(18):2598-2602.

Appendix E: Sequential life history differential gene expression analysis

Among the significant DE genes, 1,257 genes started with a statistically significant DE genes at the first time-point (i.e. stage 1: Polyp), 1,334 genes portrayed a significant linear enrichment or repression during the reverse development sequence, and 1,445 genes portrayed a significant curved response (i.e. change in linear behavior), which could indicate transitory behavior of genes. Genes in the same hierarchical cluster show similar patterns of gene expression during the reverse development sequence of *T. dohrnii*, starting with the colonial polyp (hydranth) stage and ending with the reversed polyp.

A functional gene enrichment analysis via Fischer's Exact Test of the combined 224 genes in Cluster 5 was performed to identify specific biological processes that were the enriched and suppressed in the Cyst. There was a total of 209 over- and 3 under-expressed categories within the Cyst and 74 over- and 3 under-expressed when simplified to the most specific biological category (i.e. most specific child term within a GO lineage). The reduced dataset was sorted from the most significantly differentially expressed GO term and visualized in an enrichment chart. The most specific enriched biological process in the Cluster 5 was 'Nematode larval development (GO:0002119)' and 'Positive regulation of growth rate (GO:0030307)' (Figure E1). Though *T. dohrnii* is evolutionarily distant from nematodes, comparative embryology (i.e. similarities in embryos among animals) indicates that there may be developmental networks that are similar among animals [94-96]. Additionally, the Cyst has commonalities with planulae, as both stages precede the juvenile polyp stage [97-99]. Furthermore, categories related the response to DNA damage and protein monoubiquitination were found to be highly enriched. Processes associated to cytoskeleton and chromosome

organization, both specific child-GO terms of broader mitotic cell division processes, were also found to be suppressed (Appendix F).

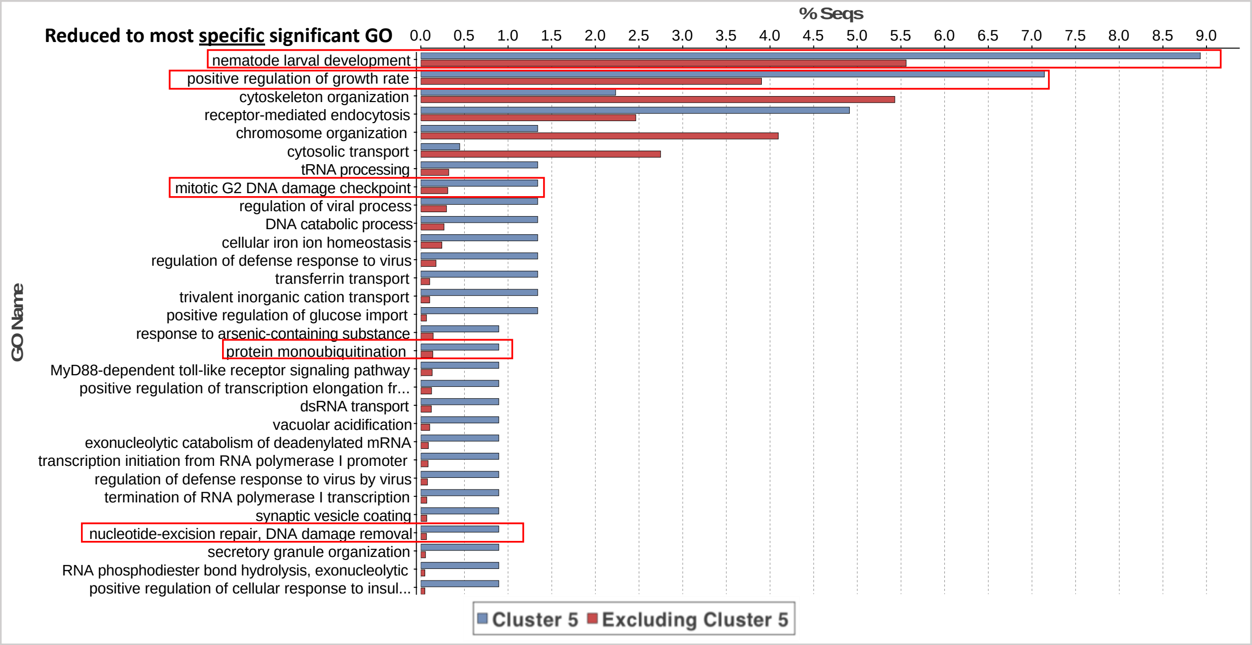

Figure E1: Functional gene enrichment analysis of Cluster 5, where significantly enriched and suppressed categories were reduced to the most specific terms (sorted by: highest in Cluster 5 or excluding Cluster 5).
