# Supplementary material for: Cellular Reprogramming and Immortality: Expression Profiling Reveals Putative Genes Involved in Turritopsis dohrnii’s Life Cycle Reversal
Source: Genome Biol Evol. 2021 Jun 16;13(7):evab136. doi: 10.1093/gbe/evab136 (PMC8480191; doi:10.1093/gbe/evab136)
Supplement: evab136_Supplementary_Data [file evab136_supplementary_data.zip › Appendix_GJ_MAY2021.pdf]

Appendix G: Pair-wise differential gene expression analyses

Polyp vs. Reversed Polyp

A total of 2,905 DE gene were identified when the Reversed Polyp was compared to the Polyp stage, where 1,622 genes were up-regulated in the Reversed Polyp stage and 1,283 were downregulated (i.e. upregulated in the Polyp stage) (Figure 5A in main text). There were substantially less DE genes in the Reversed Polyp vs. Polyp analysis in comparison to the Medusa vs. Polyp. It was expected that the Medusa and Polyp would have more differences in genetic networks and biological processes as the stages occupy completely different niches and reproductive strategies (i.e. planktonic, solitary, sexual reproduction vs. benthic, colonial, asexual reproduction), while the Reversed Polyp and Polyp are the same lifecycle stages. It was noted that there is possibility of leftover biological contaminants from fungal species in the Reversed Polyp stage that were unable to be filtered during the previous BLASTx step and may contribute to genes being up-regulated in the Reversed Polyp. Some of the enriched genes, such as reproduction and symbiotic process related-genes, may be a result of contaminants introduced from the rearing process, but genuine asexual reproduction processes or other symbiotic processes cannot be ruled out.

Medusa vs. Polyp

The Reversed Polyp libraries were not used in the comparison to aim for a more accurate analysis, as there was potential leftover fungal and contaminants that may have resulted from the culturing process. A total of 4,486 DE gene were identified when the Medusa was compared to the Polyp stage, where 2,432 genes were up-regulated in the Medusa stage and 2,053 were downregulated (i.e. upregulated in the Polyp stage). Due to the large number of DE genes, the

top 50 DE genes were used as our introductory investigation of the differences in the genetic and biological networks of the Medusa and the Polyp stage.

Appendix L: Details to methods

Transcriptome assembly

Trinity version 2.2.0 (command-line; Grabherr et al., 2011)

Trinity --max_memory 53G --CPU 20 --inchworm_cpu 6 --normalize_reads --trimmomatic --no_version_check --seqType fq -- AllReads_R1.fq --right AllReads_R2.fq

- Input: all 11 RNA-seq PE datasets pooled into single fastq file
  - k-mer size of 25 (default)
- Output: Assembled transcriptome in fasta format (PanamaTurri2019.fasta)
  - Assembly statistics generated using trinitystats.pl
  - Estimate transcript and gene abundance using align_and_estimate_abundance.pl
    - Generates align_and_estimate_abundance.txt

Transcriptome Quality Assessments

CLC Genomic Workbench v8.0.1 mapper (CLC-GUI)

Toolbox > NGS Core Tools > Map Reads to Reference

- Input: *in silico* normalized PE reads (generated from Trinity's --normalize_reads option)
- Reference dataset: Assembled transcriptome
  - No masking
- Read alignment (all default except Length/Similarity fraction)
  - Mismatch cost- 2 (linear gap cost)
    - Insertion cost: 3
    - Deletion cost: 3
  - Length fraction
    - Analysis 1 (medium)- 0.5
    - Analysis 2 (stringent)- 0.8
  - Similarity fraction
    - Analysis 1 (medium)- 0.5
    - Analysis 2 (stringent)- 0.8
  - Non-specific match handling- map randomly

BUSCO v3 with OrthoDB v9 (Ubuntu virtual machine from https://busco-archive.ezlab.org/v3/; Simão et al., 2015)

All dependencies included in virtual machine

python ~/BUSCOVM/busco3/scripts/run_BUSCO.py -i PanamaTurri2019.fa -o BUSCO_PanamaTurri2019 -l ~/BUSCOVM/lineages/metazoa_odb9 -m tran

- Input: Assembled transcriptome
- E-value cutoff (default): 0.001
- Output: short_summary_PanamaTurri2019.txt

Biological contaminant filtering

Kraken metagenomic classification tool (OmicsBox-GUI)

Metagenomics > Taxonomic classification > Kraken (Wood and Salzberg, 2014)

- Input: Assembled transcriptome (contigs)
- Database: Archaea, bacteria, viral genomes from RefSeq 2019.01
- Output: Assembled transcriptome w/ filtered contaminants via Kraken

Transcriptome annotation

Blast2GO PRO/OmicsBox annotation pipeline (OmicsBox-GUI)

OmicsBox Functional analysis module

- **BLASTx**
    - Database: NCBI's Non-Redundant (NR) v4
    - e-value cutoff: e^{-3}
    - Word size: 6
        - Low complexity filter: true
    - HSP length cutoff: 33
    - Top 20 BLAST hits saved
        - Blast description annotator: true
- **Gene Ontology (GO) Mapping and Annotation**
    - Database: GO version 2019.01
    - BLAST filter e-value cutoff: e^{-6}
    - Annotation cutoff: 55
    - GO weight: 5
    - Filter GO by taxonomy: false
- **InterProScan**
    - Databases and sequence features: CDD, HAMAP, HMMPanther, HMMPfam, HMMPIR, FPrintScan, BlastProDom, ProfileScan, HMMSmart, HMMTigr, PatternScan, Gene3D, SFLD, SuperFamily, Coils, MobiDBLite, Phobius, SignalPHMM and TMHMM.
    - Merged with BLASTx annotations
    - ANNEX Augmentation
    - Removal of 1st level GO annotations (i.e. cellular components, biological processes, molecular function)
- **EggNOG 5.0.0 (EggNOG mapper 1.0.3)**
    - Database: EggNOG 5.0.0
    - Target orthologs: All
    - GO evidence: non-electronic
    - Merged with BLASTx and IPS GO terms
        - e-value cutoff: e^{-3}
        - bit score cutoff: 60
- **KEGG GO enzyme code mapping**
    - Map against KEGG database via OmicsBox
    - Database: KEGG database
- **RFAM**
    - Blast against Xfam servers (rfam.xfam.org) via OmicsBox
    - Database: Xfam
- **BLASTn Hydrozoa EST database**
    - Database: NCBI's EST_others with taxid: 6074 (Hydrozoa) taxonomy filter
    - e-value cutoff: e^{-10}
    - Top 20 BLAST hits saved
        - Blast description annotator: true
    - Word size: 11

            - Low complexity filter: true
    - HSP length cutoff: 33
    - Subsequent BLASTx on sequences with BLASTn hits (e-value cutoff: e^{-10})

<u>Generating transcript-level estimation count data</u>
RSEM (OmicsBox-GUI; Li and Dewey, 2011))
Transcriptomics > Create count table

- Raw PE reads (11 libraries) trimmed with Phred cutoff of 10 (Williams et al., 2018)
    - In CLC Genomic Workbench v8.0.1 (CLC-GUI)
- Input: Trimmed PE reads (phred cutoff of 10) (11 libraries)
- Reference dataset: Final transcriptome assembly
    - Gene-level estimation: on
        - Transcript to gene mapping file: align_and_estimate_abundance.txt (generated with assembly via Trinity software)
- Output: .bam files of each of the 11 libraries/replicates

<u>Sequential and Pair-wise DGE</u>
Transcriptomics > Create count table > Run differential gene expression
maSigPro 1.58.0 Bioconductor package (Omicsbox-GUI; Conesa and Nueda, 2017)
Time-course expression analyses DGE

- Input: Count table (gene-level estimation) generated from RSEM
- CPM filter: 1
- Samples reaching CPM filter: 2
- Normalization method: 75% quantile
- Design type: Single series time course
- Statistical settings:
    - Significance Level (Alpha): 0.05
    - R-square cutoff: 0.7
- Visualization of results:
    - Number of clusters- 9 (default)
    - Clustering method- Hierarchical clustering
    - MDS plot generated for all 11 samples among 4 stages

edgeR
Pair-wise DGE

- Input: Count table (gene-level estimation) generated from RSEM
    - **Reversed polyp vs. polyp**
        - Simple design
        - Reference condition: Polyp
        - Contrast condition: Reversed polyp
    - **Medusa vs. polyp**
        - Simple design
        - Reference condition: Polyp
        - Contrast condition: Medusa
- CPM filter: 1
- Samples reaching CPM filter: 3

- Normalization method: TMM
- Robust: true

Functional gene enrichment analyses

FatiGO Fisher's Exact Test (OmicsBox-GUI; Al-Shahrour et al., 2004)

Functional analysis > Enrichment analysis > Enrichment analysis (Fisher's Exact Test)

- Input: Annotated final transcriptome assembly
  - Sequential (time-course)
    - Test set: ID list of Cluster 5
    - Reference set: ID list of DEGs excluding Cluster 5
  - Pair-wise
    - Test set: ID list of Reversed Polyp (Analysis 1) or Medusa (Analysis 2)
    - Reference set: Polyp (Both analyses)
  - Filter value: 0.05
  - Filter mode: FDR p-value
  - Two tailed: false
  - Annotations: GO term
    - GO Categories: Biological Processes
